# Environmental Pollution and Risk of Childhood Cancer: A Scoping Review of Evidence from the Last Decade

**DOI:** 10.3390/ijms25063284

**Published:** 2024-03-14

**Authors:** María del Pilar Navarrete-Meneses, Consuelo Salas-Labadía, Fernando Gómez-Chávez, Patricia Pérez-Vera

**Affiliations:** 1 Laboratorio de Genética y Cáncer, Instituto Nacional de Pediatría, Mexico City 04530, Mexico; mnavarretem@pediatria.gob.mx (M.d.P.N.-M.); consusa@hotmail.com (C.S.-L.); 2 Laboratorio de Microbiología Molecular, Instituto Politécnico Nacional—ENMyH, Mexico City 07738, Mexico; fgomezch@ipn.mx

**Keywords:** childhood cancer, environmental pollution, air pollution, pesticides, childhood leukemia, electromagnetic fields

## Abstract

The long-term effects of environmental pollution have been of concern as several pollutants are carcinogenic, potentially inducing a variety of cancers, including childhood cancer, which is a leading cause of death around the world and, thus, is a public health issue. The present scoping review aimed to update and summarize the available literature to detect specific environmental pollutants and their association with certain types of childhood cancer. Studies published from 2013 to 2023 regarding environmental pollution and childhood cancer were retrieved from the PubMed database. A total of 174 studies were eligible for this review and were analyzed. Our search strategy brought up most of the articles that evaluated air pollution (29%) and pesticides (28%). Indoor exposure to chemicals (11%), alcohol and tobacco use during pregnancy (16%), electromagnetic fields (12%), and radon (4%) were the subjects of less research. We found a particularly high percentage of positive associations between prenatal and postnatal exposure to indoor (84%) and outdoor (79%) air pollution, as well as to pesticides (82%), and childhood cancer. Positive associations were found between leukemia and pesticides and air pollution (33% and 27%); CNS tumors and neuroblastoma and pesticides (53% and 43%); and Wilms tumor and other rare cancers were found in association with air pollution (50%). Indoor air pollution was mostly reported in studies assessing several types of cancer (26%). Further studies are needed to investigate the mechanisms underlying the potential associations between indoor/outdoor air pollution and pesticide exposure with childhood cancer risk as more preventable measures could be taken.

## 1. Introduction

Global environmental pollution is an international public health issue with several health effects. Pollutants are harmful solids, liquids, or gases produced in unusually high concentrations that reduce the quality of the environment. Human activities have adverse effects on the environment by polluting the water, the air, and soil [1]. Large-scale human activities, such as the use of industrial machinery, power-producing stations, combustion engines, and cars, as well as field cultivation techniques, gas stations, fuel tank heaters, and cleaning procedures, influence the environment, emitting most environmental pollutants. Anthropogenic air pollution is one of the biggest health hazards worldwide, accounting for approximately 9 million deaths per year. In addition, air pollution leads to adverse effects on vegetation near factories due to exposure to heavy metals. The long-term effects of environmental pollution have been of concern as several pollutants are carcinogenic, potentially inducing a variety of cancers, including childhood cancer [1,2,3].

Childhood cancer is a leading cause of death around the world, and, thus, it is a public health issue. In many countries, this disease is the second cause of death in children over one year, exceeded only by accidents [4,5]. Approximately 280,000 children under 18 years are diagnosed with cancer each year, and it has been estimated that there will be 13.7 million new cases between 2020 and 2050 [4,5]. In children, the most frequent cancer is leukemia, accounting for 28% of the cases, followed by brain and other central nervous system tumors (26%). Other important types of childhood cancer include lymphoma, hepatoblastoma, retinoblastoma, and Wilms tumor [6,7].

Although the overall pediatric cancer incidence rate has remained steady from 2010 to 2019, it has increased since 1975, causing concern. In addition, adolescent cancer has continued to increase [6]. Important progress has been achieved regarding cure rates, and some countries report remission rates of 90–100%. However, this progress has not been achieved worldwide since some low-income countries report lower remission rates. Additionally, cure rates in adolescents have lagged behind those in children [6]. Contrasting to the enormous advances reached in cure rates, the advances in finding the causes of childhood cancer have not shown much improvement.

Even though the origins and causes of childhood cancer have not been fully understood, genetic, epigenetic, and environmental factors are recognized. It is now accepted, for childhood leukemia, that genetic aberrations potentially driving cancer can arise prenatally, and, together with secondary aberrations and epigenetic changes, the disease may develop postnatally. An important participation of infections, and how the immune system responds to them, has also been suggested as part of leukemia etiology [8,9].

Despite the abundance of research, few causes of childhood cancer have been scientifically established [4,6]. Some genetic conditions, high birth weight, and exposure to ionizing radiation are established risk factors. For many other factors, especially environmental-related ones, the scientific evidence is controversial [4]. Several epidemiological studies have suggested that environmental pollution may have a role in childhood cancer etiology. Prenatal and postnatal exposure to environmental contaminants, including air pollution, electromagnetic fields, pesticides, and radon, have been studied worldwide and are proposed as childhood cancer risk factors. However, despite the vast number of studies available, no conclusions have been drawn given the high variability of results, confounding factors, and bias [2,7,8,10,11]. In addition, the etiologies of the different childhood cancers are heterogeneous; thus, the contribution of certain factors to the development of a specific type of cancer varies. Moreover, the time of exposure can also add complexity to the environment–childhood-cancer association, as this disease has prenatal origins. Thus, additional efforts should be taken to fully clarify the relationship between environmental pollution and the risk of childhood cancer. An analysis of the available literature regarding environmental pollution and childhood cancer is helpful in order to guide the study of the potential causes of childhood cancer, which is a health issue worldwide.

The aim of the present scoping review is to analyze the available literature to detect specific environmental pollutants and their association with certain types of childhood cancer. In this study, we update and summarize the epidemiological evidence published in the last 10 years, assessing the association of prenatal and postnatal exposures to environmental factors with childhood cancer risk to push forward the investigations on the causes of childhood cancer as more preventable measures could be taken. Unlike other reviews on this topic, we have included all types of childhood cancer and not only the most frequent types of cancers; in addition, we have included prenatal exposures, adding value to our study because a broader revision was performed.

## 2. Results

**Figure 1:**
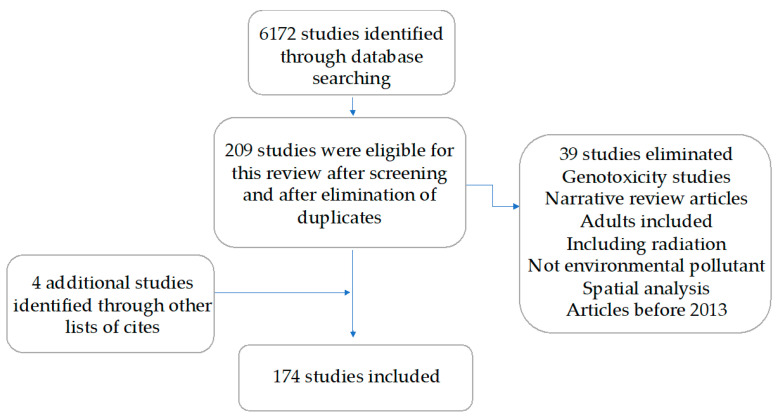
Study selection process.

A total of 6172 articles obtained from electronic MEDLINE (PuBMed) databases were retrieved, and an additional 7 were obtained from the lists of citations. Of these, 209 studies were eligible for this review because they fulfilled the inclusion criteria: (1) original or review epidemiological studies assessing the association between environmental pollution and childhood cancer; (2) published between 2013–2023; and (3) with abstracts in English or Spanish. Pollutants were categorized into the following groups: air pollution, pesticides, tobacco and alcohol exposure, indoor chemical exposure, electromagnetic fields, and radon. Studies regarding ionizing radiation from sources different from radon were excluded, as well as studies including populations other than children. Of these, 174 studies were retained after a second manual screening and elimination of studies that did not meet the inclusion criteria or were duplicated (Figure 1). Searching commands and the number of papers found in each search are described in Table 1. Table 2, Table 3, Table 4, Table 5, Table 6 and Table 7 summarize the 174 papers included in this review.

**Figure 2:**
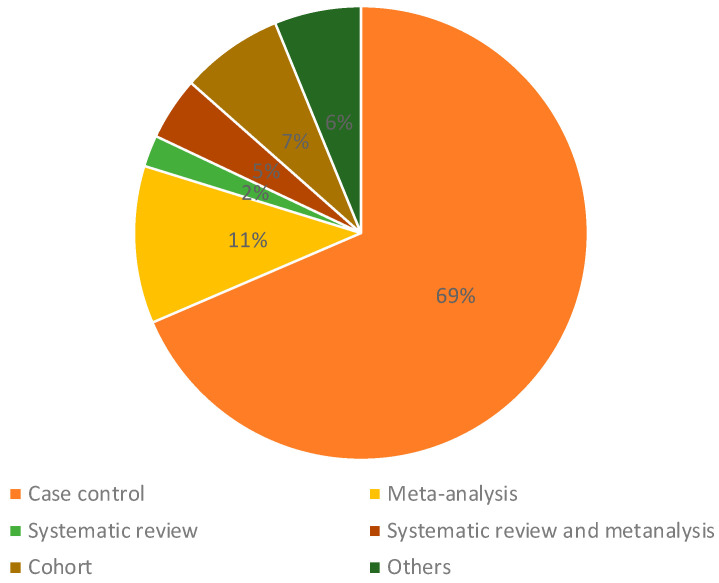
Types of studies included in this review.

**Figure 3:**
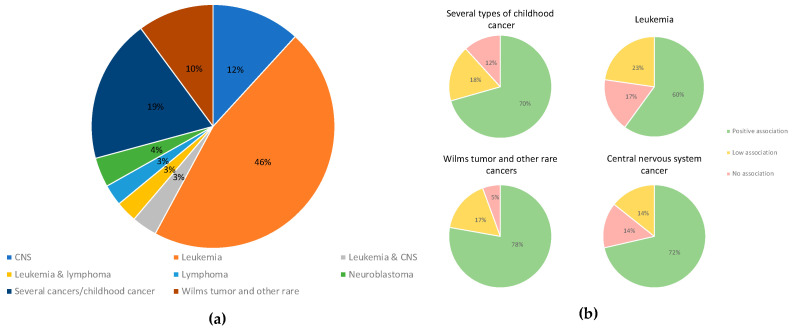
(**a**) Types of cancer included in the studies evaluated. (**b**) The smaller circles show the percentage of papers reporting positive (light green), low (light yellow), or no (light red) associations.

Most studies were case–control analyses—we found 69% of papers in this category—and 18% of studies were meta-analyses, systematic reviews, or both (Figure 2). The most investigated type of cancer was leukemia, accounting for 46% of papers reviewed. We found that 19% of the papers assessed three or more types of cancer, and 12% of papers investigated central nervous system cancer; 10% of the articles assessed Wilm’s tumor, retinoblastoma, hepatoblastoma, and other rare tumors (Figure 3). According to our categorization of results (positive association, low association, or no association with environmental pollution), the type of cancer with the most positive associations (78%) was Wilms tumor and other rare tumors, and the type of cancer with the lowest percentage of positive associations was leukemia, in which 60% of studies showed positive associations and 23% of the studies showed negative associations with environmental pollution (Figure 3). We found that 12% of studies analyzed nervous system cancer, and 72% of the studies showed positive associations with environmental pollution.

**Figure 4:**
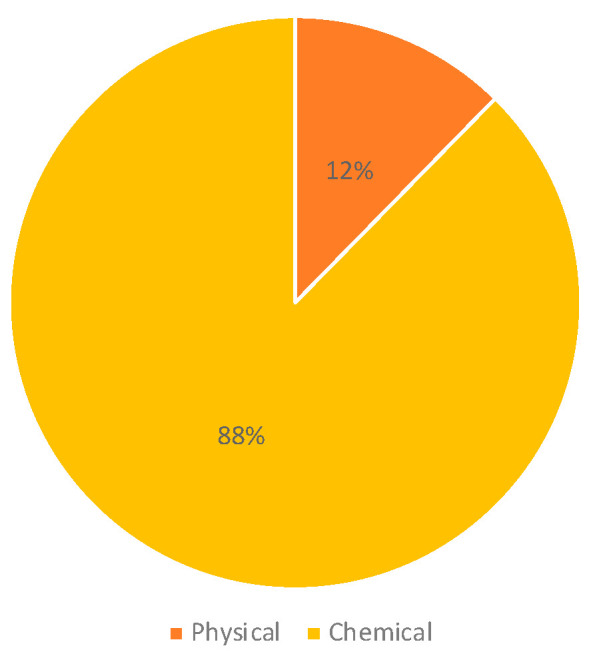
Type of pollutants included in the studies.

**Figure 5:**
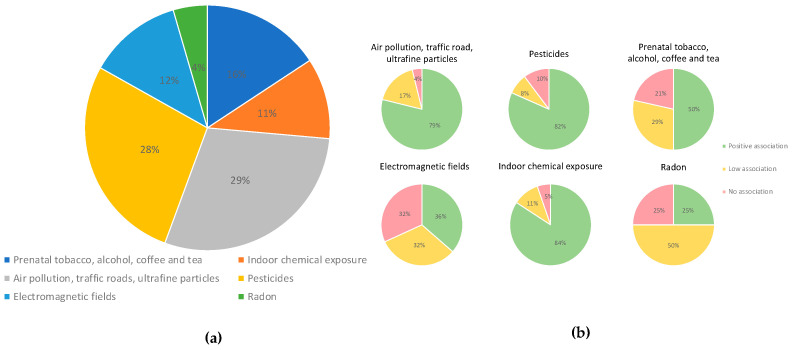
**(a**) Types of environmental pollutants analyzed in the studies included. (**b**) The smaller circles show the percentage of papers demonstrating positive (green), low (blue), or no (red) associations with environmental pollution.

Regarding the type of pollutant, we found that 88% of the investigations assessed chemical environmental factors, and 12% included physical factors such as electromagnetic fields (Figure 4). The most frequent type of environmental factor assessed was air pollution (29%), which included benzene, particulate matter, ultrafine particles, diesel engine exhaust, living in industrially contaminated sites, and living near road traffic. Studies assessing air pollution were followed by studies on pesticides (28%), papers including prenatal or parental exposure to tobacco, alcohol, coffee, and tea (16%), and papers assessing exposure to electromagnetic fields (12%); 11% of the studies assessed indoor chemical exposures (Figure 5).

The highest percentage of positive associations (84%) was found in studies assessing indoor chemical exposures, which included volatile organic compounds such as benzene, hydrocarbon solvents, air pollution at the residence (NO_2_, PM2.5), polychlorinated biphenyls, home painting, home remodeling, and others. A high percentage of positive associations was also seen among studies assessing pesticides (82%) and air pollution (79%). A lower percentage of positive associations was observed among studies assessing prenatal exposure to tobacco and alcohol (50%) and radon (25%) (Figure 5).

**Figure 6:**
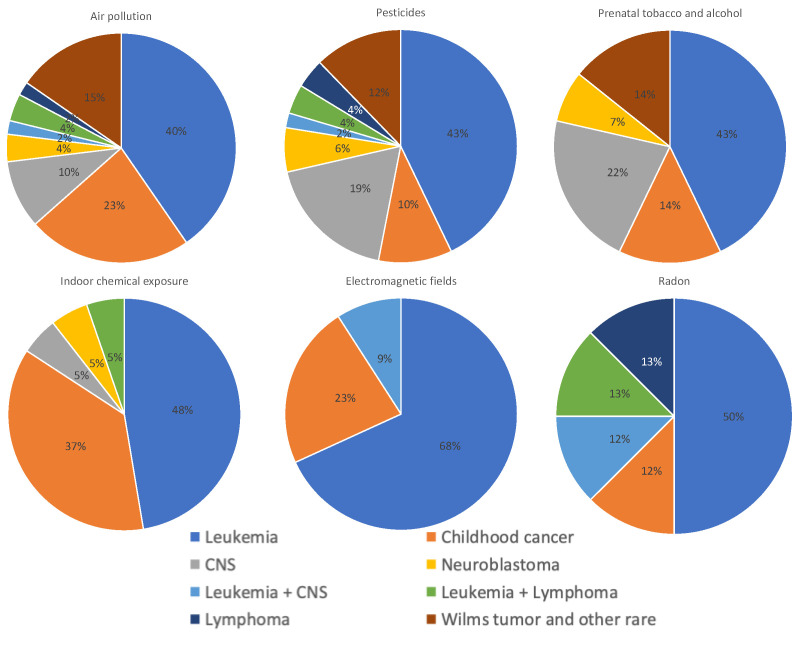
Distribution of types of cancer assessed in studies on air pollution, pesticides, parental tobacco and alcohol, indoor chemicals, electromagnetic fields, and radon exposures. All studies, those with positive, low, or negative results, were included in this analysis.

As mentioned above, the type of cancer most frequently analyzed was leukemia, ranging from 68% in studies on electromagnetic fields to 40% in studies on air pollution. For all the pollutants, studies assessing more than one type of cancer have been performed, particularly for indoor chemical exposures (37%). An exclusive analysis of childhood CNS cancer has been included, particularly in studies on prenatal tobacco and alcohol exposure and pesticides. Although no studies were found exclusively assessing Wilms tumor and other rare cancers and exposure to electromagnetic fields or radon, these types of cancers could have been included in studies that investigated more than one type of cancer (category “childhood cancer”) (Figure 6).

**Figure 7:**
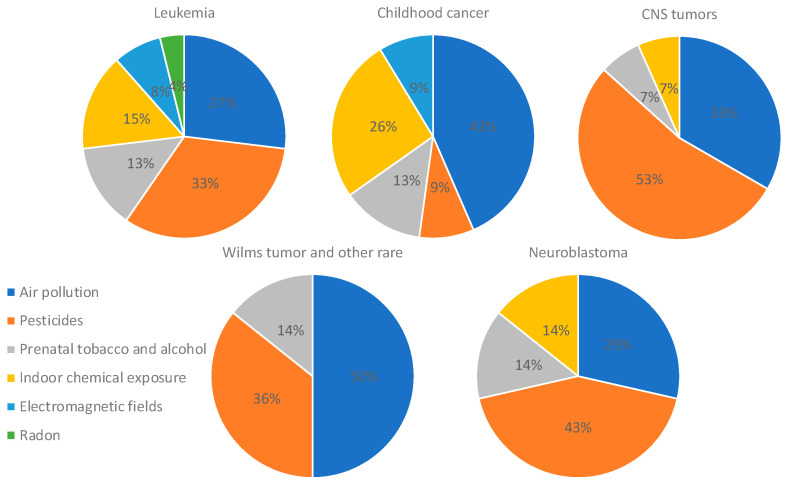
Distribution of studies showing positive associations between each type of cancer and pollutant. Only studies that found positive associations were included in this analysis.

**Figure 8:**
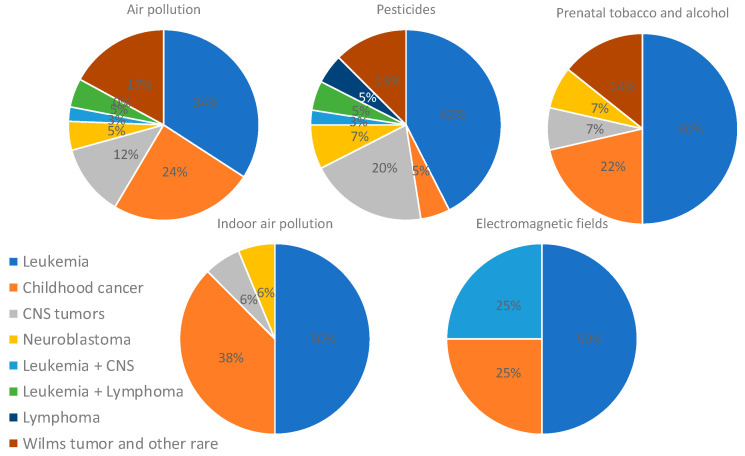
Distribution of types of cancer assessed in studies on air pollution, pesticides, parental tobacco and alcohol, indoor chemicals, and electromagnetic fields exposures. Only studies showing positive associations were included for this analysis.

An analysis focusing only on studies that found positive associations was performed, and it revealed that leukemia was mostly associated with pesticides and air pollution (33% and 27%, respectively). Studies assessing more than two types of childhood cancer found mostly an association with air pollution and indoor chemical exposure (43% and 26%), whereas studies on CNS tumors and neuroblastoma found associations with pesticides (53% and 43%). Studies assessing Wilms tumor and other rare cancers found an association mostly with air pollution (50%) (Figure 7). In addition, the analysis of only positive associations focusing on the type of pollutant showed that air pollution was associated with leukemia (34%), several types of childhood cancer (24%), and Wilms tumor and other rare cancers (17%); pesticides were associated with leukemia (42%), CNS tumors (20%), and Wilms tumor and other rare cancers (13%); prenatal tobacco and alcohol exposure was associated mostly with leukemia (50%); and indoor air pollution was associated mostly with leukemia (50%) and several types of cancer (38%) (Figure 8).

**Figure 9:**
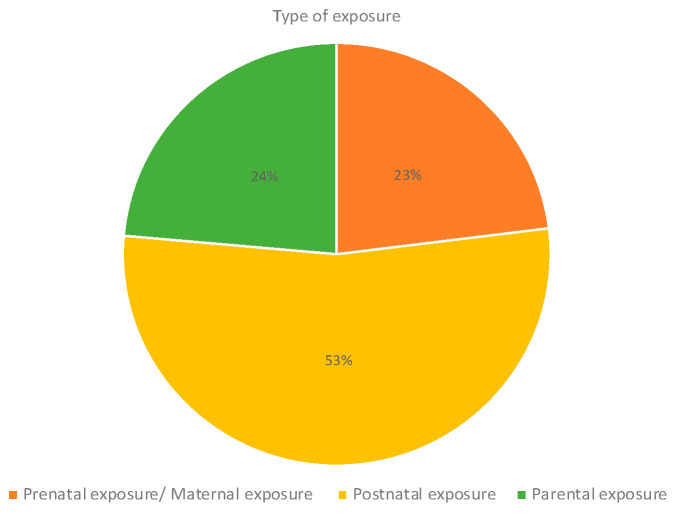
Type of exposure.

**Figure 10:**
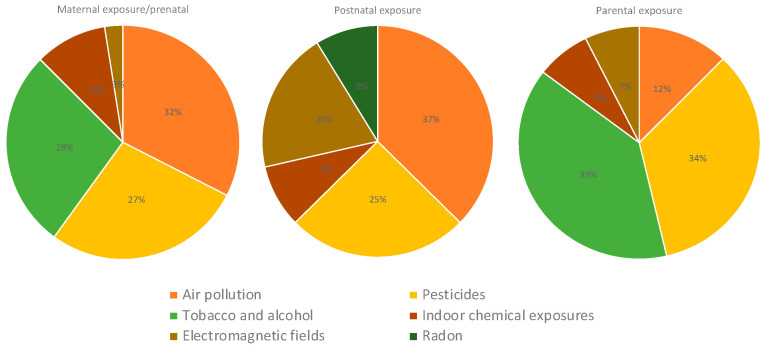
Type of exposure in studies assessing air pollution, pesticides, tobacco and alcohol, indoor chemical, electromagnetic fields, and radon exposures.

Regarding the type of exposure, most studies included postnatal exposure (53%), particularly studies assessing air pollution and pesticides (Figure 9); 23% of the studies analyzed maternal exposure to contaminants during pregnancy, particularly to air pollution, pesticides, and tobacco and alcohol exposures. Studies investigating parental exposures mostly included studies on pesticides and tobacco and alcohol consumption. Indoor chemical exposures were mostly assessed postnatally (Figure 10).

## 3. Discussion

Environmental pollution is a global public health issue, particularly for susceptible groups such as children, who are vulnerable during their development [1]. Among the multiple childhood diseases associated with environmental pollution, cancer is of concern because it is a leading cause of death for children and adolescents, and the causes have not been fully understood [8]. The analysis of the potential environmental risk factors associated with childhood leukemia is important because preventable measures could be established. In the present review, we update and summarize the epidemiological evidence published in the last 10 years, assessing the association of prenatal and postnatal exposures to environmental factors with childhood cancer risk. Although relatively recent reviews on the topic are available in the literature, in the present study, we have updated the information to 2023 and also have included prenatal studies, which have not been included in recent reviews [2]. Moreover, compared to other recent reviews, we have focused not only on childhood leukemia, but have also included all types of childhood cancer [188]. The inclusion of all types of childhood cancer, as well as the inclusion of prenatal exposures, are the strengths of this review. Overlap of original studies among systematic reviews may exist, which is a limitation of this review. Other limitations of this study are the exclusive use research in PubMed and the exclusion of papers published before 2013. Thus, the conclusions should be taken with caution. The following paragraphs discuss our main findings on each of the groups of contaminants detected.

### 3.1. Air Pollution

**Figure 11:**
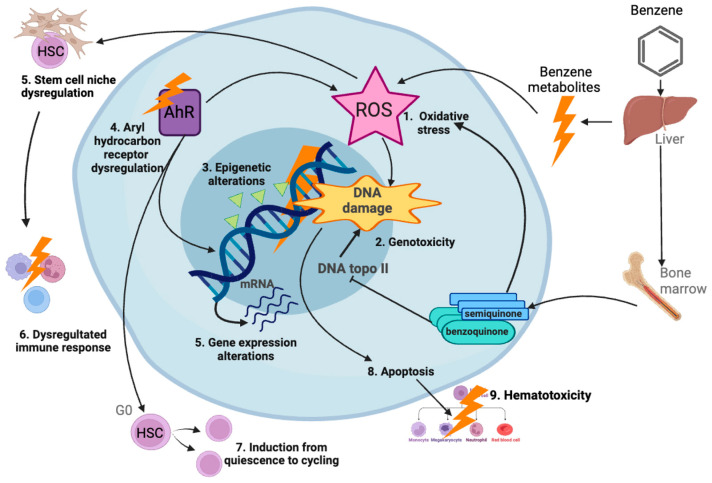
Mechanisms of benzene-induced leukemia. Benzene is metabolized in liver, lung, and bone marrow, producing metabolites which exert different biological effects, including oxidative stress inducing reactive oxygen species (ROS); genotoxicity, including mutations, chromosome breaks, and aneuploidy; and epigenetic changes, dysregulation of aryl hydrocarbon receptor (AhR), alteration of gene expression, and apoptosis. Oxidative stress can dysregulate hematopoietic stem cell (HSC) niche and lead to a dysregulated immune response. Moreover, Ahr disruption can induce HSCs from quiescence (G0) to cycling, leading to hematotoxicity. In combination, these events can lead to leukemogenesis [192]. Created with BioRender.com.

More than one quarter of the literature (29%) retrieved included air pollution. This contaminant was mostly positively associated with leukemia, and it was frequently reported in studies that evaluated several types of childhood cancer (Figure 8). In addition, we observed that studies assessing Wilms tumor and other rare cancers found an association with air pollution (Figure 7). This is the second leading cause of non-communicable diseases globally, and has been classified by IARC as a human carcinogen regarding lung cancer. In addition, particulate matter and several other components of air pollution have also been classified as carcinogenic to humans. Moreover, air pollution is the most widespread environmental carcinogen. The predominant sources of outdoor air pollution are transportation, stationary power generation, industrial and agricultural emissions, and residential heating and cooking [189,190]. In the present study we have separated the analysis of outdoor air pollution from the studies on indoor air pollution. A wide number of studies evaluated air pollution and childhood cancer risk by assessing whether living nearby heavy-traffic roads or proximity to industrial and urban sites is associated with an increased cancer risk. Numerous studies have found positive associations not only with leukemia, but also with CNS cancer, neuroblastoma, Wilms tumor, and bone cancer, among other cancers (Table 2) [13,14,16,24,29,30,31,32,34,37,43,46,54,61]. However, other studies have found mild or negative associations [12,16,21,25,39,45,60,185]. Benzene is one of the major components of air pollution and is carcinogenic to the bone marrow, causing leukemia and myelodysplastic syndromes; it is suggested that it also affects the lymphatic system, causing lymphoma. It has been reported that benzene induces genotoxic and non-genotoxic events in utero, that could potentially lead to childhood leukemia [191]. In murine models bearing preleukemic cells, benzene induced fast leukemic transformation. Benzene metabolites can induce oxidative stress, genotoxicity, epigenetic modifications, aryl hydrocarbon receptor dysregulation, gene expression alterations, and apoptosis induction; these events can lead to the dysregulation of immune response and hematotoxicity, potentially contributing to leukemogenesis (Figure 11) [192]. Although these investigations on benzene could represent a plausible mechanism underlying the association of air pollution exposure and childhood leukemia, for other types of childhood cancer with a different etiology, the mechanisms must be further explored. In countries such as Mexico, where the incidence of childhood leukemia is high, the levels of benzene have been detected above the reference concentration in the urine of children living near shoe workshops [193]. Regulations on the air concentration of benzene and other established carcinogens must be applied as the evidence clearly shows their harmful potential.

### 3.2. Pesticides

The literature on pesticides (49 articles), together with air pollution (52 articles), represents more than one-half of the studies reviewed. Positive associations were found between pesticides and leukemia, as well as CNS tumors (Figure 8). Pesticides are used worldwide in several human activities, and tons of these chemicals are produced globally. However, the health effects of these products have been of concern because they are associated with diseases such as cancer [11]. To reduce environmental pollution and toxicity, research on bio-based pesticides is crucial, in order to have alternatives to replace chemical pesticides [194]. Although their association with childhood cancer has not been fully demonstrated, we found that more than 80% of the epidemiological studies show positive associations with this disease (Table 3, Figure 5). A recent study that evaluated pesticides in the urine samples of parents and children from five European countries reported that 84% of samples showed at least two pesticides, highlighting the global exposure to these chemicals [195]. Unfortunately, most of these studies lack the characterization and quantification of pesticides in the human body, and generally rely only on self-reported uses. In addition, humans are not exposed to a single type of pesticide but to a mixture of them, adding complexity to studies on the relationship between these pollutants and childhood cancer. Worldwide, studies have been performed and have shown that prenatal and postnatal exposure to pesticides is associated with higher risks, not only of leukemia, but also of lymphoma, retinoblastoma, neuroblastoma, CNS cancer, and Wilms tumor (Table 3, Figure 8).

**Figure 12:**
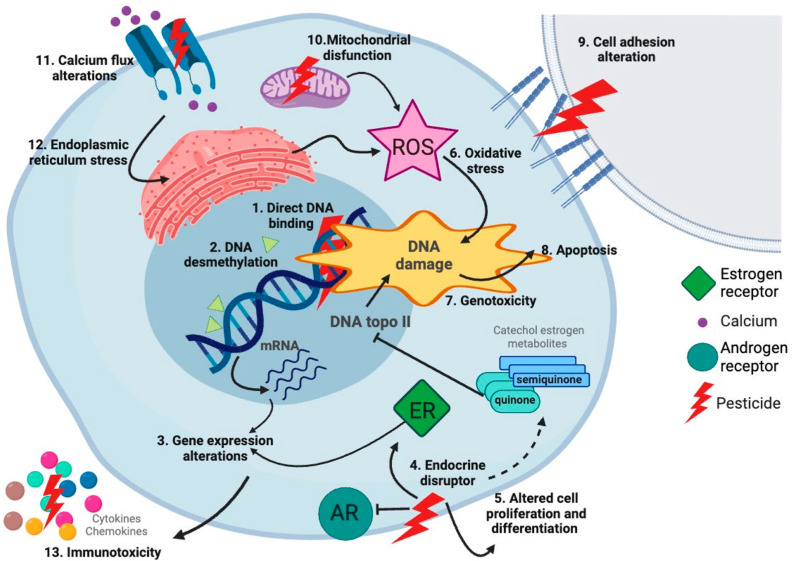
Mechanisms of pesticide-induced cancer. Pesticides such as pyrethroids exert different biological events that can potentially promote cancer. Pyrethroids can bind DNA, induce epigenetic changes, alter gene expression, be endocrine disruptors, alter cell proliferation and differentiation, induce oxidative stress increasing reactive oxygen species (ROS), be genotoxic, induce apoptosis, alter cell adhesion, disrupt mitochondrial function, alter calcium flux, and induce endoplasmic reticulum stress. All these events in combination could drive leukemogenesis [197]. Created with BioRender.com.

Different classes of pesticides have been associated with childhood cancer, including organochlorides, organophosphates, and pyrethroids (Table 3). Regulatory agencies have considered several pesticides as carcinogens, such as DDT, but, for many others, such as pyrethroids, the evidence has not been enough to be catalogued as carcinogens [196]. The mechanisms underlying the associations of pesticides with childhood cancer may be different according to the type of pesticides. For pyrethroids, which are one of the most common classes of insecticides, evidence has shown that they can induce multiple biological effects potentially linked to cancer. Pyrethroids can induce genotoxic and non-genotoxic effects, and are immunotoxins, neurotoxins, oxidative stress producers, and endocrine disruptors; all these effects could promote the initiation or development of cancer (Figure 12) [197]. Importantly, our results revealed a high percentage of studies showing associations between CNS tumors, neuroblastoma, and pesticides (Figure 7). The mechanisms assessing the potential relationship of pesticides with these types of cancer should be explored. Although the genotoxicity of pesticides has been evaluated, their epigenetic effects need to be fully investigated because their contribution to childhood cancer etiology could be at this level [198]. In addition, although the risks of pesticide exposure have been vastly evaluated, the transgenerational effects of these exposures need more attention. It has been shown that the environmentally induced disease risk can be transmitted to the offspring, via epigenetic mechanisms through female and male germ lines [199].

### 3.3. Tobacco and Alcohol

We found that 16% (28 articles) of the studies are destined to investigate the association of tobacco and alcohol exposure with childhood cancer. However, other studies could have been missed by our searching strategy, given that we did not search “tobacco” or “alcohol” intentionally. The 28 articles presented here were found with the searching strategy described in the following sections. Contrasting with pesticides and air pollution, studies on tobacco and alcohol showed a lower percentage of positive associations. Only 50% of the studies showed a clear association with childhood cancer (Table 4, Figure 5), showing that the association of these pollutants with childhood cancer is even more contradictory. Studies suggest an association of tobacco and alcohol exposure with leukemia, CNS cancer, retinoblastoma, neuroblastoma, and Wilms tumor, particularly during the prenatal period. Although tobacco use accounts for 25% of all cancer deaths globally, and is the primary cause of lung cancer in adults, its role in childhood cancer is less clear [200]. Efforts to reveal whether tobacco and alcohol exposures are related to childhood cancer are needed because these exposures are completely preventable. It is known that cigarettes contain dozens of carcinogens, including benzene, whose leukemogenic potential has already been discussed [201].

### 3.4. Indoor Chemical Exposure

Studies on chemical exposure at home accounted for 11% of the articles included. For the purposes of this review, indoor exposure was separated from outdoor exposure. However, several of the pollutants are shared between indoor and outdoor exposures, including NO_2_, benzene, and VOCs; in addition, chemical exposures at home may include paints and other solvents used to clean or carry out home remodeling (Table 5). As expected, as seen with air pollution analysis, a high number of studies showing positive associations between indoor chemical exposures and childhood cancer were observed (Figure 5 and Figure 8). The mechanisms underlying the role of indoor chemical exposure and childhood cancer are expected to be like the mechanisms supporting the associations with outdoor air pollution exposures. We have to consider that the concentrations of chemicals indoors and outdoors might be different. Efforts to inform people of the risks of using certain chemicals at home, such as paints, as well as measures helping to improve indoor air quality, such as correct ventilation or optimal cooking practices, should be improved, particularly in homes with pregnant women and children [7].

### 3.5. Electromagnetic Fields

Our search strategy retrieved 22 articles that investigated electromagnetic fields and childhood cancer, which represented 12% of the studies included. Given the global expansion in wireless networks, concern has been raised regarding the possible health effects of low-to-mid-frequency electromagnetic fields (LM-EMFs). LM-EFs include extremely low-frequency EMFs (EL-EMFs) which are produced by power lines, electrical wiring, and electrical appliances; and radiofrequency EMFs, which mostly come from wireless telecommunication devices, including cell phones, tablets, and laptop computers [202]. Contrasting to higher-frequency EMFs which includes X-rays and gamma rays and are in the ionizing radiation part of the electromagnetic spectrum, LM-EFs are not known to damage DNA or cells directly. However, other potential cellular effects of LM-EFs have been of concern [203]. The studies retrieved by our search showed a low number of positive associations between EMFs and childhood cancer (36%) (Figure 5), showing a higher controversy of results compared to other environmental risk factors. The studies assessing EMFs mostly included childhood leukemia (Figure 6), showing that further studies are needed to evaluate other specific types of cancer. Although some studies have shown that prenatal and postnatal exposure to EMFs can increase the risk of childhood leukemia (Table 6), the most recent studies have shown mild to no associations [158,159,160].

### 3.6. Radon

Few studies regarding radon exposure and childhood cancer were retrieved by our search strategy (8 studies). Importantly, we did not include “radon” in the search strategy. Possibly, other studies, including radon and childhood cancer, were missed by our strategy. Ionizing radiation exposure at therapeutic doses was beyond the scope of this review as it is a well-established childhood cancer risk factor. Natural radiation comes from radon exposure, and its relationship with childhood cancer is unclear. Although studies have suggested that domestic radon exposure is associated with higher childhood leukemia risk [180,181], others have shown mild to no association, neither with childhood cancer nor with other cancers such as lymphoma and CNS cancer [102,183,185,186,187]. Thus, the possible role of radon in childhood cancer remains controversial and needs further investigation.

Compared to previous recent reviews, some coincidences have been detected in our analysis. Regarding childhood leukemia, an umbrella review in 2021 found convincing evidence of an association between general prenatal pesticide exposure and the risk of this type of cancer; this result was also retrieved by our study. In the same study, the authors found some level of evidence of an association of EMFs, benzene, indoor air pollution, and prenatal tobacco exposure with childhood leukemia. Our results coincide with this previous study except for EMF exposure, because our study retrieved mostly low or negative results for EMFs. However, this needs to be taken with caution as few articles regarding EMFs were included in this study [188]. A more recent review of the same author confirmed the convincing evidence of the pesticide exposure association with acute lymphoblastic leukemia risk, and found little evidence for radon association with this cancer [204]. In addition, a 2021 scoping review of environmental risk factors and all types of childhood cancer mostly found articles regarding air pollution, chemical exposures, radiation, and residential locations. Our study agrees with finding a great number of articles regarding air pollution and chemical exposures. However, the previously reported scoping review did not include prenatal exposures, and pesticide exposures were included in the category of chemical exposures together with other types of chemicals [2].

Our results show that several studies have found a positive association between certain types of cancer and specific pollutants. Given its highest frequency, most pollutants were expected to be associated with childhood leukemia. We observed that, together, indoor and outdoor air pollution add up to 42% of the studies assessing causes of childhood leukemia and showing positive results, and pesticides represented 33%. Regarding CNS tumors and neuroblastoma, 53% and 43% of the studies, respectively, presented positive associations with pesticides. Studies that analyzed more than two types of childhood cancer found associations with indoor and outdoor air pollution (69%). Although epidemiological data strongly suggest associations of air pollution and pesticides with childhood cancer, particularly leukemia and SNC tumors, more mechanistic evidence is needed. Studies are required to demonstrate that prenatal or postnatal exposure to these chemicals can promote biological events associated with leukemia and brain tumor etiology, such as specific gene mutations, epigenetic changes, the modification of signaling pathways, increased proliferation, and escape from apoptosis. In utero studies with animal models are particularly valuable, given the prenatal origin of childhood cancer. Additionally, analyses of exposomes from cancer patients and controls are valuable for detecting specific levels of chemicals included in the global groups of “air pollution” and “pesticides”. Research on the effects of individual chemicals is important, but it is also needed for evaluating how all these molecules interact with each other and collaborate in promoting childhood cancer, which could be achieved through AI models. On the other hand, the epidemiological evidence regarding electromagnetic fields is still controversial, and mostly negative results were found in our review. Thus, more epidemiological studies are needed regarding electromagnetic field exposure and childhood cancer risk.

This review shows that, in particular, outdoor and indoor air pollution and pesticides are associated with childhood cancer in numerous studies, which could help guide the research into the causes of childhood cancer. In addition, regulatory measures could be considered based on these results. For example, the re-evaluation of specific contaminants is necessary in order to update their classifications as carcinogens. Additionally, more regulatory measures are needed to improve the air quality in countries like Mexico City, which has a history of high environmental pollution. Furthermore, disseminating information and education to the global population on the safe use of chemicals at home is important in order to avoid high exposure indoors. Regulatory measures are urgently needed to prevent childhood cancer, which is a global health issue with a worryingly increasing incidence. It is important to highlight that this review shows limitations such as the overlap of original studies and systematic reviews; therefore, the conclusions should be taken with caution. The strengths of this review are the inclusion of all types of childhood cancer because most reviews focus only on specific types of cancer, and the inclusion of prenatal exposures which has also been eliminated in some review studies. Prenatal exposures are important given the intrauterine origin of childhood cancer. It is hypothesized that genetic alterations may arise in utero, and, later in life, this could drive carcinogenesis, if secondary mutations arise along with additional epigenetic events. Detecting environmental exposures that could induce prenatal genetic alterations is important for the prevention of childhood cancer.

## 4. Materials and Methods

### 4.1. Data Source and Search Strategy

An extensive peer-reviewed original epidemiological search of MEDLINE (PuBMed) database was conducted in January and June 2023 to identify studies regarding childhood cancer and environmental factors. Three researchers participated independently in the search. Studies included were limited to studies on humans. The following search strategy was applied: “child” OR “childhood” AND “cancer” AND “pollution”, “pesticides”, “magnetic fields”, “benzene”, “smoke”, “water pollution”, and “air pollution”. Studies regarding radiation and childhood cancer were not included in this study as radiation is a well-established cause of childhood cancer. However, exposure to indoor radon was included as controversy still exists. Searching was performed through titles and abstract screening, and, when needed, additional information was obtained from the main article. Reference lists of articles were examined for additional relevant literature.

### 4.2. Study Selection

Criteria for inclusion and exclusion were defined previously. We included articles published between 2013 and 2023. No geographical or language restrictions were applied. However, when the information of interest was not available in abstracts, studies in languages other than English or Spanish were excluded. Studies selected included case–control studies, cohort studies, ecological studies, systematic reviews, and meta-analyses assessing environmental exposure and childhood cancer. Exposure times included prenatal and childhood time windows. Studies assessing parental exposures were also included. Risk assessment studies, in vitro and in vivo assays, and narrative, scoping, or umbrella reviews were not included but were evaluated for additional literature. Duplicates were eliminated using Zotero 6.0.32.

### 4.3. Data Extraction

For each included article, we recorded the first author’s name and year of publication, type of childhood cancer included, type of pollutant investigated, country, study design, main outcomes and results, and number of participants. Data were summarized in a table.

### 4.4. Data Analysis

A second review was performed to eliminate articles that did not meet all the criteria described in Section 4.2. Data were organized on graphics containing the type of environmental factor, type of cancer analyzed, type of study, type of exposure, and type of association. Each outcome was evaluated and categorized as “positive association”, when a clear and significant association between the risk factor and childhood cancer was reported; “low association” when results did show some association but were taken with caution because confounding factors and bias were not excluded; and “no association” when results did not show an association between the investigated risk factor and childhood cancer.

## 5. Conclusions

Environmental pollution and childhood cancer are both worldwide health issues that deserve considerable attention. A vast amount of literature shows that childhood cancer risk may be related to environmental prenatal and early childhood exposures. According to the findings of this scoping review, prenatal and postnatal exposures to indoor and outdoor air pollution and to pesticides seem to be positively associated with childhood cancer risks, including leukemia, CNS cancer, Wilms tumor, and other rare childhood cancers, given the high percentage of studies showing positive associations. There is some evidence linking radon and electromagnetic field exposure to pediatric cancer; however, the correlation is not strong, given the large amount of research demonstrating weak or negative relationships. However, some limitations of this review must be considered, including the overlap of original studies across systematic reviews.

Further studies are needed to investigate the mechanisms underlying the potential associations between air pollution, pesticide exposure, and childhood cancer risk. Determining the specific air pollution components, as well as the group of pesticides, related to childhood cancer etiology and their mechanisms could help to establish better regulatory preventive measures, and to detect vulnerable populations. Besides genotoxicity and carcinogenicity studies, investigations assessing other potential biological effects, including epigenetic modifications, are necessary. Additionally, studies assessing the potential transgenerational effects of these pollutants regarding childhood cancer are needed.

## Figures and Tables

**Table 1:** Number of articles retrieved by each searching command.

Searching Commands	Number of Papers Retrieved
“pollution” “childhood” “cancer”	323
“children” “cancer” “pollution”	1788
“children” “cancer” “pesticides”	376
“children” “cancer” “magnetic” “fields”	172
“children” “cancer” “benzene”	98
“smoke” “children” “cancer”	2121
“childhood” “cancer” “water” “pollution”	17
“childhood” “cancer” “air” “pollution”	187
“childhood” “cancer” “pollution”	324
“childhood” “cancer” “pollutants”	766

**Table 2:** Studies assessing air pollution and childhood cancer risk.

Reference	Pollutant	Cancer	Population	Results OR 95% CI	Type of Study
Zhong, C., 2023 [12]	Air pollution and artificial light at night (ALAN)	ALL	California	ALAN 1.15, 1.01–1.32; PM2.5 1.24, 0.98–1.56	Large population-based case–control: 2782 ALL cases and 139,100 controls
Malavolti, M., 2023 [13]	Residential proximity to petrol stations	Leukemia	Italy	RR was 2.2, 0.5–9.4 for children living <50 m from the nearest petrol station. Associations were stronger for the ALL subtype RR 2.9, 0.6–13.4.	Population-based case–control: 182 cases and 726 controls
Kreis, C., 2022 [14]	Traffic-related air pollution	ALL	Switzerland	HR NO_2_ and ALL 1.00, 0.88–1.13; AML 1.31, 1.00–1.71; benzene and ALL 1.03, 0.86–1.23; AML 1.29, 0.86–1.95	Cohort study: 2960 cases
Lee, 2022 [15]	Particulate matter	Childhood cancer	Korea	HR PM2.5 3.02, 1.63-5.59	Retrospective cohort: 1725 patients
Mazzei, 2022 [16]	Residential proximity to petrol stations and benzene	Childhood cancer	Switzerland	All cancers 1.29, 0.84–1.98; Leukemia 1.08, 0.46–2.51; CNS 1.30 [0.51–3.35]	Case–control: 6129 cases
Asenjo, S., 2022 [17]	Cadmium and lead topsoil levels	Leukemia	Spain	A unit increase on the topsoil level for cadmium and leukemia 1.11, 1.00–1.24; for lead and leukemia 1.10, 0.99–1.21	Case–control: 2897 cases
Onyije, 2021 [18]	Residential proximity to petroleum facilities	Leukemia	France	Petroleum and childhood leukemia, summary effect size [ES] 1.90, 1.34–2.70	Systematic review and meta-analysis
Ribeiro, A., 2021 [19]	Residential traffic exposure	Lympho hematopoietic	Sao Paulo	Lymphoid leukemia and traffic density, IRR 1.21, 1.06–1.39 and 1.3, 1.13–1.68 for NO_2_ for the lower SES group. In the higher group, 1.06, 1.00–1.14 traffic density and 1.37, 1.16–1.62 for NO_2_	Retrospective population-based registry
Lavigne, E., 2020 [20]	Ultrafine particle concentrations [UFP]	Childhood cancer	Toronto	First trimester exposure to UFPs per 10,000/cm^3^ increases HR 1.13, 1.03–1.22 cancer.	Population-based cohort: 1066 cases
Hvidtfeldt, 2020 [21]	Residential exposure to PM2.5 components	Non-Hodgkin’s lymphoma	Denmark	PM2.5, 2.05, 1.10–3.83 and for BC, 1.22, 1.02–1.46, SIA 1.73, 0.68–4.41, and NHL	Case–control: 170 cases
Volk, J., 2019 [22]	Parental occupational exposure to diesel engine exhaust	Leukemia and CNS	Denmark	Maternal exposure to diesel engine and CNS cancer 1.31, 0.99–1.74, and astrocytoma 1.49, 1.04–2.14	Register-based nested case–control: 1673 cases
Peckham-Gregory, 2019 [23]	Maternal residential proximity to major roadways	Leukemia	Texas	No association mothers who lived ≤500 m to a major roadway and ALL No association mothers who lived in areas characterized by high roadway density and ALL or AML	Case–control study: 2030 cases
García-Pérez, 2019 [24]	Vicinity of pollution sources	Childhood cancer	Spain	Proximity [≤2 km] to specific industrial areas and leukemias 1.31, 1.04–1.65, and 1.28, 1.00–1.53 for urban areas, and neuroblastoma 2.12, 1.18–3.83 for both industrial and urban areas, and renal 2.02, 1.16–3.52 for industrial areas	Population-based case–control
Gong, 2019 [25]	Traffic-related air pollution	Leukemia		Traffic density 1.01, 0.98–1.04, high traffic density 1.04, 0.91–1.17, moderate exposure to NO_2_ 1.02, 0.93–1.10, and benzene 1.04, 0.71–1.37, with risk of leukemia	Meta-analysis
Filippini, 2019 [26]	Outdoor air pollution	Leukemia		Traffic density RR 1.09, 1.00–1.20; Benzene 1.27, 1.03–1.56; NO_2_ 1.04, 0.90–1.19; PM2:5 1.05 0.94–1.16; PM10 1.20, 0.70–2.04; 1,3—Butadiene 1.45, 1.08–1.95 with leukemia	Systematic review and dose–response meta-analysis
Seifi, M., 2019 [27]	Exposure to ambient air pollution	Childhood cancer	Tehran, Iran	Cancer and PM10 1.008, 1.001–1.015; NO2 1.05, 0.98–1.01	Case–control: 161 cases
Hall, C., 2019 [28]	Prenatal exposure to air toxics	Malignant germ cell tumors	California	Prenatal exposure to 1,3-butadiene during the second trimester and GCT, 1.51, 0.01–2.26 and meta/para-xylene 1.56, 1.10–2.21]. Elevated ORs for yolk sac tumors.	Case–control: 243 cases
Iavarone, 2018 [29]	Living in industrially contaminated sites	Childhood cancer	Italy	Contaminated areas, especially of industrial origin and CNS cancer in the age-group <1 year SIR 3.2, 1.4–6.3; soft tissue sarcoma in the age-group 0-14 years SIR 1.6, 1.1–2.3; AML in the age group 0-14 years SIR 1.7, 1.1–2.4;	Cohort: 1050 cases
Kirkeleit, 2018 [30]	Exposure to gasoline exhaust	Leukemia	Norway	Gasoline or exhaust and increased risk of leukemia HR 2.59, 1.03–6.48 and ALL HR 2.71, 0.97–7.58	Prospective population-based cohort study
Kumar, S., 2018 [31]	Maternal residential proximity to major roads	Childhood cancer	Texas	Embryonal tumor in children born to mothers living within 500 m of a major roadway 1.24, 1.00–1.54. For unilateral retinoblastoma 2.57, 1.28–5.15, for every kilometer closer the mother lived to the nearest major roadway.	Case–control
Ortega-García, 2017 [32]	Proximity to air-polluting industries	Childhood cancer	Spain	SNST was around energy-generating chemical industries, another of NHL was around residue-valorization plants, and, finally, one cluster of HL was around building materials	Retrospective cohort
Janitz, A.E., 2017 [33]	Benzene	Leukemia	Oklahoma	Cases born from 2005 to 2010 had a three-fold increased unadjusted odds of elevated exposure to benzene and leukemia compared to controls born in this same time 3.53, 1.35–9.27.	Case–control
Ramis, R., 2017 [34]	Exposure to industrial/urban environment	CNS	Spain	Children living in industrial and urban areas and CNS tumors. Urban areas 0.90, 0.65–1.24, and industrial areas 0.96, 0.81–1.77	Case–control: 714 cases
Lavigne, E., 2017 [35]	Maternal exposure to ambient air pollution	Childhood cancer	Ontario	PM2.5, over the entire pregnancy, and during the first trimester and astrocytoma HR per 3.9 μg/m^3^ 1.01–1.88, and HR per 4.0 μg/m^3^ = 1.40, 1.05–1.86, respectively. First trimester NO_2_ and ALL HR 1.20, 1.02-1.41 per IQR [13.3 ppb].	Population-based: 2044 cases
Spycher, D., 2017 [36]	Parental occupational exposure to benzene	Childhood cancer	Switzerland	Maternal exposure to benzene and leukemia HR 1.73, 1.12–2.67, and ALL 1.88, 1.16–3.04.	Case–control: 1520 cases
García-Pérez, 2017 [37]	Residential proximity to industrial and urban areas	Bone tumors	Spain	Close to industrial facilities as a whole 2.33, 1.17–4.63, at 3 km. Surface treatment of metals 2.50, 1.13–5.56, at 2 km; production and processing of metals 3.30, 1.41–7.77, at 2.5 km; urban waste–water treatment plants 4.41, 1.62–11.98, at 2 km; hazardous waste 4.63, 1.37–15.61, at 2 km; disposal or recycling of animal waste 4.73, 1.40–15.97, at 2 km; cement and lime 3.89, 1.19–12.77, at 2.5km; and combustion installations 3.85, 1.39–10.66, at 3 km, and bone tumors	Case–control: 114 cases
Janitz, E., 2017 [38]	Maternal and paternal occupational exposures	Hepatoblastoma	USA	Both paternal and maternal exposure to paints and hepatoblastoma: paternal 1.71, 1.04–2.81; maternal 3.29, 0.32–33.78].	Case–control: 383 cases
Janitz, A., 2016 [39]	Traffic-related air pollution	Leukemia	Oklahoma	NO_2_ [11.19–19.89 ppb] and AML with a positive [4th quartile] 5.25, 1.09–25.26	Case–control: 307 cases
Danysh, E., 2016 [40]	Maternal residential proximity to major roadways	CNS	Texas	Mothers living ≤500 m from a major roadway were 31%, 1.0–1.8 more likely to have offspring with any CNS tumor and 3.1-times 0.9–10.4 more likely to have offspring with an ependymoma compared to mothers living >500 m from the nearest major roadway. Comparing mothers living in areas with low roadway density vs. areas with high roadway density were 51%, 1.1–2.1 more likely to have offspring with any CNS tumor and 4.2-times 1.2–14.9 more likely to have offspring with an ependymoma.	Case–control: 315 cases
Von Ehrenstein, O., 2016 [41]	In utero and early-life exposure to ambient air toxics	Brain tumors	California	Prenatal exposure to acetaldehyde and primitive neuroectodermal tumors 2.30, 1.44–3.67, 1,3-butadiene 2.23, 1.28–3.88, and exposure during the first year of life to ortho-dichlorobenzene 3.27, 1.17–9.14, 1,3-butadiene 3.15, 1.57–6.32. Medulloblastoma was associated with prenatal exposure to polycyclic aromatic hydrocarbons 1.44, 1.15–1.80. Exposures to lead and some PAHs during the first year of life and astrocytoma 1.40, 0.97–2.03.	Population-based case–control: 183 cases
Symanski, E., 2016 [42]	Air toxics	ALL	Texas	Polycyclic organic matter and leukemia, 1.11, 0.94–1.32, 1.17, 0.98-1.39 for benzene, and 1.29, 1.08–1.52 for 1,3-butadiene.	Population-based case–control
Magnani, C., 2016 [43]	Road traffic pollution	Leukemia	Italy	Closeness of the house to traffic lights and to the passage of trucks [Road traffic pollution] and ANLL 6.35, 2.59–15.6.	Nationwide case–control: 648 cases
García-Pérez, 2016 [44]	Residential proximity to environmental pollution	Renal tumors	Spain	Children living near [≤2.5 km] industrial installations and renal tumors 1.97, 1.13–3.42.	Case–control: 213 cases
García-Pérez, 2016 [45]	Residential proximity to environmental pollution	Rare tumors	Spain	Retinoblastoma and proximity to industries involved in glass and mineral fibers 2.49, 1.01–6.12 at 3 km, and organic chemical industries 2.54, 1.10–5.90 at 2 km.	Population-based case–control: 557 cases
García-Pérez, 2016 [46]	Residential proximity to industrial and urban sites	Neuroblastoma	Spain	Neuroblastoma and intersection between industrial and urban areas: 2.52, 1.20–5.30 for industrial distance of 1 km, and 1.99, 1.17–3.37 for industrial distance of 2 km.	Population-based case–control: 398 cases
Carlos-Walace, 2016 [47]	Parental, in utero and early-life exposure to benzene	Leukemia		Occupational and household product exposure and RR leukemia was 1.96, 1.53, 2.52. RR was higher for AML 2.34, 1.72–3.18 than for ALL 1.57; 1.21–2.05. Traffic density or traffic–related air pollution RR 1.48, 1.10–1.99; it was higher for AML 2.07, 1.34–3.20 than for ALL 1.49, 1.07–2.08	Meta-analysis
Filippini, T., 2015 [48]	Outdoor air pollution	Leukemia		NO_2_ and benzene 1.21, 0.97–1.52 and 1.64, 0.91–2.95 respectively. Stratifying by leukemia type, upon NO_2_ were 1.21, 1.04–1.41 for ALL, and 1.06, 0.51–2.21 for AML; upon benzene were 1.09, 0.67–1.77 for ALL, and 2.28, 1.09–4.75 for AML.	Meta-analysis
Spycher, B., 2015 [49]	Residential exposure to highways	Childhood cancer	Switzerland	Comparing children living <100 m from a highway with unexposed children [≥500 m] was 1.43, 0.79–2.61. Associations were similar for ALL RR 1.64, 1.10–2.43, and stronger for leukemia in children aged <5 years RR 1.92, 1.22–3.04.	Cohort
Malagoli, C., 2015 [50]	Living in urban areas	Leukemia	Italy	Benzene and PM10 and leukemia, associated with residence in a highly urbanized area and residential area 1.4, 0.8–2.4 and 1.3, 0.8–2.2, respectively.	Case–control: 111 cases
Heck, J., 2015 [51]	Exposure to toxics in perinatal period	Retinoblastoma	California	Pregnancy exposure to benzene and retinoblastoma 1.67, 1.06–2.64. Pregnancy exposure to chloroform 1.35, 1.07–1.70, chromium 1.29, 1.04–1.60, para–dichlorobenzene 1.24, 1.04–1.49, nickel 1.4, 1.08–2.01, and, in the first year of life, acetaldehyde 1.62, 1.06–2.48 and retinoblastoma	Case–control: 103 cases
Houot, J., 2015 [52]	Residential proximity to heavy-traffic roads	Leukemia	France	A 300 m increase in major road length within 150 m of the geocoded address and AML 1.2, 1.0–1.4.	Case–control: 2760 cases
Greenop, K., 2015 [53]	Vehicle refuelling, use of domestic wood heaters	Brain tumors	Australia	Paternal refueling ≥ 4 times/month and risk of CBT 1.59, 1.11–2.29. Wood heaters before 1.51, 1.05–2.15 and after 1.44, 1.03–2.01 the child’s birth and risk of CBT.	Case–control: 306 cases
García-Pérez, 2015 [54]	Residential proximity to industrial and urban sites	Leukemia	Spain	Leukemia associated with living near [≤2.5 km] industries 1.31, 1.03–1.67—particularly glass and mineral fibers 2.42, 1.49–3.92, surface treatment using organic solvents 1.87, 1.24–2.83, galvanization 1.86, 1.07–3.21, production and processing of metals 1.69, 1.22–2.34, and surface treatment of metals 1.62, 1.22–2.15.	Population-based case–control: 638 cases
Zhou, 2014 [55]	Maternal benzene exposure during pregnancy	ALL		Solvent 1.25, 1.09–1.45; paint 1.23, 1.02–1.47; petroleum exposure 1.42, 1.10–1.84, and maternal smoking during pregnancy 0.99, 0.93–1.06 associated with ALL	Meta-analysis
Shrestha, A., 2014 [56]	Prenatal exposure to air toxics	Wilms tumor	California	Prenatally exposed to formaldehyde, polycyclic aromatic hydrocarbons, perchloroethylene, or acetaldehyde in the third trimester and Wilms’ tumor per interquartile increase in concentration 1.28, 1.12–1.45; 1.10, 0.99–1.22; 1.09, 1.00–1.18; 1.25, 1.07–1.45, respectively.	Case–control: 337 cases
Boothe, V., 2014 [57]	Residential traffic exposure	Leukemia		Residential traffic exposure and leukemia [postnatal exposure window] 1.53, 1.12–2.10	Systematic review and meta-analysis
Heck, J., 2014 [58]	Exposure to ambient toxics during pregnancy and early childhood	Leukemia	California	Polycyclic aromatic hydrocarbons [third trimester exposure] and ALL 1.16, 1.04–1.29; arsenic 1.33, 1.02–1.73; benzene 1.50, 1.08–2.09; AML associated to chloroform [third trimester exposure] and 1.30, 1.00–1.69; benzene 1.30, 1.00–1.69	Case–control: 69 cases
Badaloni, C., 2013 [59]	Air pollution	Leukemia	Italy	All ORs, independent of the method of assessment and the exposure windows, were close to the null value.	Nationwide case–control: 620 cases
Heck, J., 2013 [60]	Traffic-related air pollution	Childhood cancer	California	Traffic–related pollution during the first trimester and ALL 0.05, 1.01–1.10; germ cell tumors 1.1, 1.04–1.29, particularly teratomas 1.26, 1.12–1.41; and retinoblastoma 1.11, 1.01–1.21, particularly bilateral retinoblastoma 1.16, 1.02–1.33	Case–control: 3590 cases
Ghosh, J., 2013 [61]	Prenatal exposure to traffic-related air pollution	Childhood cancer	California	ALL and higher air pollution exposures by 9% per 25 ppb increase in nitric oxide 1.09, 1.01–1.18. For every 25 ppb increase in third-trimester pollutant concentrations, increase risk of bilateral retinoblastoma 15%, 1.01–1.31, and 13%, 0.99–1.29 for nitric oxide and nitrogen dioxide, respectively.	Case–control: 4015 cases
Peters, S., 2013 [62]	Occupational exposure to engine exhausts	Brain tumors	Australia	Maternal exposure to diesel exhaust and brain tumors any time before the child's birth 2.03, 1.09–3.81 and paternal exposure around the time of the child’s conception 1.62, 1.12–2.34.	Population-based case–control: 306 cases
Heck, E., 2013 [63]	Ambient air toxics exposure in pregnancy	Neuroblastoma	California	Maternal exposure to carbon tetrachloride and neuroblastoma 2.65, 1.07–6.53, and polycyclic aromatic hydrocarbons [indeno[1,2,3-cd], pyrene and dibenz[a,h]anthracene] 1.39, 1.05–1.84. Hexavalent chromium and neuroblastoma 1.32, 1.00–1.74, at 5 km.	Case–control: 75 cases

OR 95% CI: odds ratio 95% confidence interval; ALAN: air pollution and artificial light at night; PM2.5: particulate matter smaller than 2.5 microns; ALL: acute lymphoblastic leukemia; HR: hazard ratio; AML: acute myeloid leukemia; CNS: central nervous system; UFP: ultrafine particle concentrations; BC: black carbon; SIA: secondary inorganic aerosols; NHL: non-Hodgkin’s lymphoma; PM10: particulate matter smaller than 10 microns; GCT: germ cell tumors; SNST: sympathetic nervous system tumor; PAH: polycyclic aromatic hydrocarbons; ANLL: acute non-lymphoblastic leukemia; RR: relative risk; CBT: childhood brain tumor; HCS: hydrocarbon solvents; EEF: engine exhaust fumes; ML: myeloid leukemia; HL: Hodgkin’s lymphoma; STS: soft tissue sarcoma; AHS: Agriculture Health Study; PCP: pentachlorophenol; RM: ratio of means; RMS: rhabdomyosarcoma; PCB: polychlorinated biphenyls; PBDES: polybrominated diphenyl ethers; ELF-MF: extremely low-frequency magnetic fields; RMF: residential magnetic fields; HVOL: high-voltage. Colors indicate the degree of associations: positive [green], low [yellow], or no [red] associations.

**Table 3:** Studies assessing exposure to pesticides and childhood cancer risk.

Reference	Pollutant	Cancer	Population	Results OR 95% CI	Type of Study
Ward, 2023 [64]	Pesticides [glyphosate]	ALL	California	No association of occupational pesticide exposure, and ALL	Case–control: 181 cases and 225 controls
Rafeeinia, 2023 [65]	Pesticides [organochlorines]	ALL	Iran	76.2% of CDKN2B promoters, and 85.1% of MGMT promoters were hypermethylated in children with ALL	Case–control: 72 cases and 141 controls
Rossides, M., 2022 [66]	Maternal and paternal exposure to pesticides	Childhood cancer	Sweden	Aromatic HSC and N-HL 1.64, 1.05–2.58; aliphatic/alicyclic HCS and GCT 1:52, 0.89–2.59; gasoline/diesel EEF and astrocytoma 1:40, 1.04–1.88, ML 1:53, 0.84–2.81, HL 1:60, 0.85–3.02, EEF and HL 1:21, 1.01–1.44; STS 1:22, 1.00–1.48	Large population-based case–control: 17,313 cancer cases
Thompson, S., 2022 [67]	Prenatal exposure to pesticides	Retinoblastoma	California	EEF and HL 1:21, 1.01–1.44; STS 1:22, 1.00–1.48	Case–control study: 335 cases and 123,166 controls
Khan, 2022 [68]	Prenatal exposure to pesticides	Neuroblastoma		Prenatal pesticide exposure and neuroblastoma 1.6, 1.1–2.3, while after birth, 1.0, 0.8–1.3.	Systematic review and meta-analysis
El-Helaly, 2022 [69]	Pesticides, radiation, hazardous chemicals, and smoking	Bone cancer	Egypt	Nitrose compounds among children, paternal smoking, and consanguinity are predictors of bone cancer.	Retrospective case–control study: 51 cases and 67 controls
Khan, 2022 [70]	Pesticides	Wilms tumor		Parental pesticide exposure and Wilms’ tumor. A strong association between organophosphate herbicides/insecticides and pediatric cancer was found.	Meta-analysis and systematic review
Bamouni, 2022 [71]	Residential proximity to croplands at birth	Leukemia	France	No association between croplands density and acute leukemia.	Case–control: 8747 cases and 19,809,700 controls
Onyije, 2022 [72]	Parental exposure to pesticides and other agents	Leukemia	Europe [UK, France, Spain, Lithuania, Norway, and Greece]	High paternal occupational exposure to crystalline silica and ALL 2.20, 1.60–3.01, and for AML 2.03, 1.04–3.97. For ALL, high paternal occupational exposure to chromium 1.23, 0.77–1.96, and diesel engine exhaust 1.21, 0.82–1.77.	Case–control study: 3362 cases and 6268 controls
Feulefack, 2021 [73]	Prenatal exposure to pesticides	Brain cancer		Pesticides prenatal exposure and brain tumors 1.32; 1.17–1.49. After birth exposure 1.22, 1.03–1.45, and residential exposure to pesticides 1.31, 1.11–1.54. Parental occupational exposure and CBT 1.17, 0.99–1.38.	Meta-analysis
Nguyen, 2021 [74]	Proximity of residence at birth to outdoor plant nurseries	Leukemia	USA	For birth residences less than 75 m from plant nurseries and leukemia 2.40, 0.99–5.82; stronger for AML 3.09, 1.14–8.34.	Case–control: 5788 cases and 5788 controls
Madrigal, 2021 [75]	Pesticides	ALL	USA	No association of permethrin, chlorpyrifos, diazinon, and carbaryl with ALL.	252 cases and 306 controls
Coste, A., 2020 [76]	Parental occupational exposure to pesticides	Childhood cancer	Switzerland	No association with maternal or paternal exposure to pesticides and any cancer subtype.	1891 cases of cancer
Patel, 2020 [77]	Parental occupational exposures to pesticides, animals, and organic dust	Leukemia and CNS	Australia, Denmark, Israel, Norway, and UK	Paternal exposures to pesticides and animals and risk for AML herbicides HR = 3.22, 0.97–10.68; insecticides 2.86, 0.99–8.23; animals 3.89, 1.18–12.90. Paternal exposure to organic dust and AML 2.38, 1.12–5.07.	329,658 participants from birth cohorts in five countries with ALL (129), AML (31), and CNS tumors (158)
Park, 2020 [78]	Pesticide exposure in pregnancy	Leukemia	USA	Exposure to any carcinogenic pesticide and risks for ALL 2.83, 1.67–4.82, diuron 2.38, 1.57–3.60, phosmet 2.10, 1.46–3.02, kresoxim-methyl 1.77, 1.14–2.75, and propanil 2.58, 1.44–4.63. Elevated risks for the group of 2,6-dinitroanilines 2.50, 1.56–3.99, anilides 2.16, 1.38–3.36, and ureas 2.18, 1.42–3.34.	Case–control: 162 cases and 9805 controls
Mavoungou, 2020 [79]	Pesticide exposure during pregnancy	Lymphoma	France	Insecticides association with Burkitt lymphoma and mixed cellularity classical HL 1.3, 1.0–1.7.	Case–control: 328 cases with Hodgkin’s lymphoma, 305 with non-Hodgkin’s lymphoma, and 2415 controls
Rios, 2020 [80]	Pesticides and other agents	Wilms tumor	France	Maternal use of pesticide during pregnancy associated with the risk of Wilms tumor 1.6, 1.1–2.3. Association was stronger when they were used more often than once a month 1.9, 1.2–3.0.	Case–control: 117 cases and 1100 controls
Coste, 2020 [81]	Agricultural crop density	Leukemia	France	Viticulture density and the incidence of ALL, SIRR = 1.03, 1.00–1.06.	Registry of cases: 11,487 cases
Patel, 2020 [82]	Residential proximity to crops and animals during pregnancy	Leukemia and CNS	Denmark	Mothers with increasing crop area near their home and leukemia highest tertile > 24 ha HR: 2.0, 1.02–3.8; after adjustment for animals [within 1000 m] HR: 2.6, 1.02–6.8. We also observed increased risk for grass/clover highest tertile > 1.1 ha HR: 3.1, 1.2–7.7, peas > 0 HR: 2.4, 1.02–5.4, and maize > 0 HR: 2.8, 1.1–6.9 in animal-adjusted models.	Case–control
Van Maele-Fabry, 2019 [83]	Domestic pesticide exposure	Leukemia		Association between residential pesticide exposure and leukemia SOR: 1.57, 1.27–1.95.	Meta-analysis
Bunch, 2019 [84]	Paternal occupational exposure to pesticides and other agents	Lymphoma	Great Britain	Paternal exposure to ceramics and/or glass and lymphoma risk both before and after adjustment for social class 2.33, 1.19–4.59 and 2.45, 1.22–4.95, respectively; specifically for both HL and NHL 4.00, 1.08–22.09 and 2.80, 1.01–7.77, respectively. BL risk and paternal exposure to lead 2.67, 1.24–5.74.	Case–control
Georgakis, 2019 [85]	Instrument-assisted delivery and other agents including pesticides	Brain tumors	Greece	Instrument-assisted delivery and risk of brain tumors. Maternal alcohol consumption during pregnancy 2.35, 1.45–3.81, and history of living in a farm 4.98, 2.40–10.32 and brain tumors.	Case–control
Hyland, 2018 [86]	Maternal residential pesticide uses before and after chld´s birth	ALL	Costa Rica	Maternal insecticide uses inside the home in the year before pregnancy, during pregnancy, and while breastfeeding associated with ALL among boys 1.63, 1.05–2.53, 1.75, 1.13–2.73, and 1.75, 1.12–2.73, respectively.	Case–control
Ferri, 2018 [87]	Parental occupations, pesticide use, environmental factors, and genetic polymorphism [CYP2D6*4]	Leukemia	Italy	Prenatal maternal use of insecticides/rodenticides 1.87, 1.04–3.33, with subjects living <100 m from pesticide-treated fields 3.21, 1.37–7.53, and with a paternal occupation as traffic warden/policeman 4.02, 1.63–9.87, and increased risk for leukemia. Associations were found with genetic polymorphism of CYP2D6*4 for homozygous alleles [mutant type/mutant type 6.39, 1.17–34.66].	Case–control
Vidart d’Egurbide Bagazgoïtia, 2018 [88]	Maternal residential use of pesticides	Brain tumors	France	Brain tumors with the maternal home use of pesticides during pregnancy 1.4, 1.2–1.8, more specifically, with insecticide 1.4, 1.2–1.8.	Case–control
Boffetta, 2018 [89]	Permethirn	Childhood cancer		An increased risk of multiple myeloma was found among AHS members with the highest tertile of estimated permethrin exposure 5.01, 2.41–10.42.	Systematic review
Van Maele-Fabry, 2017 [90]	Residential, household, and domestic exposure to pesticides	Brain tumors		Associations were observed with CBT after combining all studies with exposure to non-agricultural pesticides 1.26, 1.13–1.40.	Meta-analysis
Gunier, 2017 [91]	Parental occupational pesticide exposure from the year before pregnancy to the child's third year of life	ALL	USA	Increased risk of ALL for paternal occupational exposure to any pesticides 1.7, 1.2, 2.5, diagnosed before five years of age 2.3, 1.3, 4.1.	Case–control
Erjaee, 2017 [92]	Pesticides and other agents related to cancer	Childhood cancer	Iran	Association between the patients’ allergy [mostly food allergy] and obtaining a malignancy 2.09, 1.04–4.22. Parental smoking and risk of malignancy 1.56, 0.73–3.36. Living near high-voltage electricity lines 2.12, 1.17–3.84: Lymphoma [11.8%] osteosarcoma [9.8%], neuroblastoma [6.7%], and ALL [6.4%]. Contact with domestic animals [mostly hens, roosters, and sheep] 2.24, 1.43–3.50: adrenocortical tumors [75%], hepatoblastoma [50%], brain tumors [37%], rhabdomyosarcoma [28.6%], and ALL [28%]. Exposure to chemical pesticides and fertilizers 2.27, 1.26–4.08: adrenocortical tumors [50%], hepatoblastoma [40%], brain tumor [22%], and Ewing’s sarcoma [20%].	Case–control
Rios, 2017 [93]	Maternal use of household pesiticides during pregnancy	Neuroblastoma in mother’s offspring	France	Maternal use of any type of pesticide during pregnancy was associated with neuroblastoma 1.5, 1.2–1.9, most commonly insecticides 1.4, 1.1–1.9 or with other pesticides 2.0, 1.1–3.4.	Case–control study
Omidakhsh, 2017 [94]	Parental pesticide exposure	Retinoblastoma	USA	Unilateral retinoblastoma associated with parental insecticide use 2.8, 1.1–6.7; professional lawn or landscape services 2.8, 1.0–8.2.	Case–control
Febvey, 2016 [95]	Parental pesticide exposure	CNS	France, Germany, and the UK	Around conception, paternal occupational pesticide exposure and CNS tumors 0.71, 0.53 to 0.95.	Case–control
Gómez-Barroso, 2016 [96]	Pesticides	Childhood cancer	Spain	Living in the proximity of cultivated land could be associated with many types of cancer.	Population-based case–control
Malagoli, 2016 [97]	Passive exposure to agricultural pesticides	Leukemia	Italy	Residing close to arable crops and leukemia 2.04, 0.50–8.35, and such excess risk was further enhanced among children aged <5 years.	Case–control
Chen, 2016 [98]	Pyrethoid metabolites	Brain tumors	China	trans-DCCA, 3-PBA, and total metabolites associated with risk of CBT 2.58, 1.38–4.80; 3-PBA 3.26, 1.73–6.14; total metabolites 3.60, 1.87–6.93. Exposure to both mosquitocide and cockroach killer was related to the increased risk of CBT [mosquitocide, 1.68, 1.06–2.67; cockroach killer 1.83, 1.13–2.95, respectively].	Case–control
Chen, 2015 [99]	Residential childhood pesticide exposures and childhood cancers	[Leukemia and lymphoma]		Exposure to indoor residential insecticides was associated with risk of leukemia 1.47, 1.26–1.72, and lymphomas 1.43, 1.15–1.78. Leukemia was associated with herbicide exposure 1.26, 1.10–1.44.	Meta-analysis
Maryam, 2015 [100]	Pesticides	Leukemia	Iran	Increased risk for farmers 14.7, 5.6–38.4 and for fathers of the pediatric cases 5.4, 3.0–9.9.	Case–control
Zhang, 2015 [101]	Household exposure to pesticides	Leukemia	China	The household use of mosquito repellent and AL 1.9, 1.2–3.1.	Case–control: 248 cases and 111 controls
Chen, 2015 [102]	Pesticides and other agents related to cancer	Leukemia	China	Chemical exposure during childhood 4.76, 1.34–16.89, maternal exposure to chemicals 4.51, 1.65–12.33, household insecticides use during 0–3 years of child 2.90, 1.31–6.39, and renovating after their children's birth 3.12, 1.26–7.74 were associated with an increased risk of AL	Case–control: 66 cases
Bailey, 2015 [103]	Pesticides	Leukemia	Childhood Leukemia International Consortium	ALL associated with any pesticide exposure shortly before conception, during pregnancy and after birth were 1.39, 1.25–1.55, 1.43, 1.32–1.54, and 1.36, 1.23–1.51, respectively. Risk of AML were 1.49, 1.02–2.16, 1.55, 1.21–1.99, and 1.08, 0.76–1.53, respectively.	Case–control
Zheng, R., 2015 [104]	Occupational exposure to pentachlorophenol	Lymphoma Leukemia		Lymphoma and workers' occupational exposing to PCP 2.57, 1.52–4.35.	Systematic review
Kunkle, 2014 [105]	Farm-related pesticide exposures	Brain tumors.		CBT and farm-related exposures during pregnancy RR 1.48, 1.18–1.84. CBT and maternal exposure to non-agricultural pesticides [e.g., home extermination, pest strips] during pregnancy RR 1.36, 1.10–1.68, and risk of CBT and agricultural activities RR 1.32, 1.04–1.67. Paternal exposure to pesticides during preconception 2.29, 1.39–3.78.	Meta-analysis
Kumar, 2014 [106]	Exposure to pesticides and other agents related to cancer especially during pregnancy	Leukemia	India	Leukemia with mother's education [*p* = 0.001], occupation [*p* = 0.0005], and pesticides exposure [*p* = 0.005] during pregnancy were found. However, there were no significant links with maternal age [*p* = 0.090], history of fetal loss [0.85], history of radiography during pregnancy [*p* = 0.400], history of drug intake [*p* = 0.689], and infection [*p* = 0.696] during pregnancy.	Case–control: 132 cases
Bailey, 2014 [107]	Parental occupational pesticide exposure	Leukemia		Maternal pesticide exposure during pregnancy and the risk of ALL was 1.01, 0.78–1.30, and for paternal exposure around conception 1.20, 1.06–1.38. For AML, for maternal exposure during pregnancy was 1.94, 1.19–3.18, and for paternal exposure around conception 0.91, 0.66–1.24.	13 case–control studies
Van Maele-Fabry, G., 2013 [108]	Parental occupational exposure to pesticides [mostly farm/agricultural jobs]	Brain tumors		Statistically significant association between parental occupational exposure to pesticides and brain tumors.	Meta-analysis
Ferreira, 2013 [109]	Pesticide exposure during pregnancy	Leukemia	Brazil	Pesticides during pregnancy and ALL 2.10, 1.14–3.86, and AML 5.01, 1.97–12.7 in children 0–11 months of age, and with ALL 1.88, 1.05–5.23 at 12–23 months of age. Maternal exposure to permethrin, for children 0–11 months of age 2.47, 1.17–5.25 for ALL; and 7.28; 2.60–20.38 for AML. Maternal pesticide exposure related to agricultural activities 5.25, 1.83–15.08 for ALL, and 7.56, 1.83–31.23 for AML.	Case–control: mothers of 252 cases
Greenop, 2013 [110]	Exposure to pesticides before pregnancy, during pregnancy, and during childhood	Brain tumors	Australia	Professional pest control treatments in the home in the year before the index pregnancy, during the pregnancy, and after the child’s birth were 1.54, 1.07–2.22, 1.52, 0.99–2.34, and 1.04, 0.75–1.43, respectively. Treatments exclusively before pregnancy and during pregnancy were 1.90, 1.08–3.36, and 1.02, 0.35–3.00, respectively. Paternal occupational exposure in the year before the child’s conception was 1.36, 0.66–2.80.	Case–control: 374 cases
Metayer, 2013 [111]	Herbicides	ALL	USA	ALL and dust levels of chlorthal; for the first, second, and third tertiles were 1.49, 0.82–2.72, 1.49, 0.83–2.67, and 1.57, 0.90–2.73, respectively.	Case–control: 269 cases
Abdolah, 2013 [112]	Paternal occupational exposure to pesticides and other agents	Retinoblastoma	USA	Paternal pesticide exposure in the 10 years prior to conception 1.64, 1.08–2.50, as well as in the year before conception 2.12, 1.25–3.61. An increased risk was also observed for non-welding metal exposure during the 10 years prior to conception in the full 1.35, 0.86–2.12.	Case–control: 198 cases

OR 95% CI: odds ratio 95% confidence interval; PM2.5: particulate matter smaller than 2.5 microns; ALL: acute lymphoblastic leukemia; HR: hazard ratio; AML: acute myeloid leukemia; CNS: central nervous system; UFP: ultrafine particle concentrations; BC: black carbon; SIA: secondary inorganic aerosols; NHL: non-Hodgkin’s lymphoma; PM10: particulate matter smaller than 10 microns; GCT: germ cell tumors; SNST: sympathetic nervous system tumor; PAH: polycyclic aromatic hydrocarbons; ANLL: acute non-lymphoblastic leukemia; RR: relative risk; CBT: childhood brain tumor; HCS: hydrocarbon solvents; EEF: engine exhaust fumes; ML: myeloid leukemia; HL: Hodgkin’s lymphoma; STS: soft tissue sarcoma; AHS: Agriculture Health Study; PCP: pentachlorophenol; RM: ratio of means; RMS: rhabdomyosarcoma; PCB: polychlorinated biphenyls; PBDES: polybrominated diphenyl ethers; ELF-MF: extremely low-frequency magnetic fields; RMF: residential magnetic fields; HVOL: high-voltage. Colors indicate the degree of associations: positive [green], low [yellow], or no [red] associations.

**Table 4:** Studies assessing exposure to tobacco and alcohol and childhood cancer risk.

Reference	Pollutant	Cancer	Population	Results OR 95% CI	Type of Study
Wimberly, 2023 [113]	Prenatal exposure to alcohol, tobacco, and illicit drugs	Childhood cancer	USA	Prenatal illicit drug associated intracranial embryonal tumors PR 1.94, 1.05–3.58, including medulloblastoma PR 1.82, and supratentorial primitive neuroectodermal tumors PR 2.66, and retinoblastoma PR 3.11, 1.20–8.08. Moderate to heavy alcohol consumption and non-Hodgkin’s lymphoma PR 5.94, 1.84–19.21.	Case-only: 3145 families
Xu, K., 2021 [114]	Prenatal tobacco smoke exposure	ALL	California	Association between DNA methylation at AHRR CpG cg05575921 and B-ALL [284 cases] with a ratio of means [RM] 1.31, 1.02–1.69. Polyepigenetic smoking score was positively associated with B-ALL [482 cases], RM 1.31, 1.09–1.57	Case-only: 482 B ALL
Alyahya, M., 2020 [115]	Parental smoking behavior	Childhood cancer	Jordan	Women who had past exposure to smoke were more likely to have a child with cancer, 2.9	Case–control: 200 cases
Frederiksen, 2020 [116]	Tobacco smoking	Leukemia	Costa Rica	Paternal smoking before conception, during pregnancy, and after birth was associated with an increased risk of AML 2.51, 1.21–5.17; 3.21, 1.56–6.60; and 2.83, 1.36–5.90, respectively.	Population-based case–control: 252 ALL and 40 AML
Rios, 2020 [80]	Parental habits in the perinatal period	Wilms tumor	France	Maternal use of any type of pesticide during pregnancy was associated with the risk of Wilms tumor 1.6, 1.1–2.3. Insecticides 1.7, 1.1–2.6. The association was stronger when they were used more often than once a month 1.9, 1.2–3.0.	Case–control: 117 cases
Doganis, D., 2020 [117]	Maternal lifestyle characteristics	Wilms tumor	Greece	Positive association 5.31, 2.00–14.10 was found for mothers who consumed alcohol only before pregnancy and Wilms tumor.	Systematic review and meta-analysis
Medina-Sanson, 2020 [118]	Tobacco smoking and pesticides	ALL	Mexico	Gene-environment interaction analysis showed that NAT2 rs1799929 TT genotype confers high risk to ALL under exposure to fertilizers, insecticides, hydrocarbon derivatives, and parental tobacco smoking.	Case–control: 478 cases
Cao, Y., 2020 [119]	Paternal smoking before conception and during pregnancy	ALL		RR for smoking before conception [RR = 1.15, 95% confidence interval: 1.04–1.27] and during pregnancy [RR = 1.20, 95% confidence interval: 1.12–1.28]	Systematic review and meta-analysis
Chunxia, D., 2019 [120]	Tobacco smoke exposure	ALL, AML		Paternal smoking with ALL 1.15, 1.038–1.275. Paternal daily smoking and AML 1.242, 1.031–1.496.	Meta-analysis
Rios, P., 2019 [121]	Parental smoking, and maternal alcohol consumption during pregnancy	Neuroblastoma	French	Both parents reported having smoked during pregnancy 1.5, 1.1–2.1.	Case–control: 357 cases
Kessous, R., 2019 [122]	Smoking during pregnancy	Childhood cancer	Israel	Maternal smoking during pregnancy and increased risk for benign tumors HR 2.5, 1.57–3.83.	Population-based cohort study
Milne, E., 2018 [123]	Maternal consumption of coffee and tea during pregnancy	ALL	Childhood Leukemia International Consortium	No association was seen with ‘any’ maternal coffee consumption during pregnancy and ALL	Case–control: 2553 cases for maternal coffee and 2982 cases for tea intake
J de Smith, A., 2017 [124]	Prenatal and early-life tobacco smoke	Leukemia	California	Deletions per case was positively associated with tobacco smoke exposure, in particular, for maternal ever-smoking RM 1.31, 1.08–1.59, maternal smoking during pregnancy 1.48, 1.12–1.94, and during breastfeeding RM 2.11, 1.48–3.02. The total number of deletions was also associated with DNA methylation at the AHRR epigenetic biomarker RM 1.32, 1.02–1.69.	Case-only: 559 cases
Tettamanti, G., 2016 [125]	Maternal smoking during pregnancy	Brain tumor	Sweden	Positive associations were found among 5–9-year-old children. In this age interval, maternal smoking during pregnancy and increased risk of brain tumors RR 1.50, 0.96–2.34, astrocytoma in male RR 2.00, 1.02–3.91, and female children RR 1.80, 0.85–3.82.	Population-based cohort: 1039 cases
Vienneau, D., 2016 [126]	Prenatal exposures	Brain tumors	Denmark, Sweden, Norway, and Switzerland	Little evidence of prenatal exposures and brain tumor.	Multinational case–control
Azary, S., 2016 [127]	Parental smoking and alcohol consumption	Retinoblastoma	USA	Maternal smoking before and during pregnancy contributed to unilateral retinoblastoma: year before pregnancy 8.9; 1.5–51; month before or during pregnancy 3.3, 0.5–20.8.	Case–control: 488 cases
Metayer, C., 2016 [128]	Parental tobacco smoking	AML		Smoking during pregnancy and ALL in Hispanics 2.08, 1.20–3.61. Paternal lifetime smoking 1.34, 1.11–1.62.	Meta-analysis
Chu, 2016 [129]	Maternal smoking during pregnancy	Neuroblastoma		Possible association between maternal smoking during pregnancy and risk of neuroblastoma 1.28, 1.01–1.62.	Meta-analysis
Orsi, L., 2015 [130]	Parental smoking, maternal alcohol, coffee, and tea consumption during pregnancy	Leukemia	France	Pre-conception paternal smoking and ALL 1.2, 1.1–1.5, and AML 1.5, 1.0–2.3. High consumption of coffee [>2 cups/day] was significantly associated with ALL 1.3, 1.0–1.8.	Case–control: 747 cases
Momen, 2015 [131]	Exposure to maternal smoking during pregnancy	Childhood cancer	Danish	Increased risk of cancer among children whose mothers reported smoking cessation in pregnancy 1.46, 1.01–2.10.	Prospective, national-registry-based
Mattioli, 2014 [132]	Tobacco smoke	Leukemia	Italy	Paternal smoke in the conception period was associated with ANLL for ≥11 cigarettes/day = 1.79, 1.01–3.15.	Case–control: 82 cases
Grufferman, S., 2014 [133]	Parental military service, Agent Orange exposure	Rhabdomyosarcoma	USA	Paternal exposure to AO and RMS 1.72, 0.55–5.41	Case–control: 319 cases
Farioli, A., 2014 [134]	Tobacco smoke exposure	ALL	Italy	No association of parental active smoking and ALL	Large population-based case–control: 602
Greenop, 2014 [135]	Maternal consumption of coffee and tea during pregnancy	Brain tumors	Australia	Among children aged under 5 years, any coffee consumption during pregnancy was 1.7, 1.09–2.84, and, for ≥2 cups per day during pregnancy, was 2.52, 1.26–5.04, and brain tumors.	Case–control: 293 cases
Huang Yi, 2014 [136]	Parental smoking	Brain tumors		Maternal smoking during pregnancy, paternal smoking during pregnancy, maternal smoking before pregnancy, and paternal smoking before pregnancy and brain tumors 0.96, 0.86–1.07, 1.09, 0.97–1.22, 0.93, 0.85–1.00, and 1.09, 1.00–1.20, respectively.	Systematic review and meta-analysis
Barrington-Trimis, 2013 [137]	Parental smoking and functional polymorphisms in polycyclic aromatic hydrocarbon metabolism genes	Brain tumor	USA	A dose–response pattern for paternal smoking was observed among children with the EPHX1 H139R high-risk genotype 1.0; OR [≤3 h/day] = 1.32, 0.52–3.34; OR [>3 h/day] = 3.18, 0.92–11.0 and brain tumors.	Population-based case–control: 202 cases
Metayer, C., 2013 [138]	Tobacco smoke exposure	ALL AML	California	Paternal prenatal smoking combined with postnatal passive smoking and risk of ALL 1.01–2.23. This joint effect was seen for B-cell precursor ALL with t[12;21] 2.08. Similarly, child’s passive smoking was associated with an elevated risk of AML with chromosome structural changes 2.76, 1.01–7.58.	Case–control: 767 cases
Milne, E., 2013 [139]	Parental smoking	Brain tumors	Australia	No association of parental smoking before or during pregnancy with CBT risk.	Case–control: 302 cases

OR 95% CI: odds ratio 95% confidence interval; PM2.5: particulate matter smaller than 2.5 microns; ALL: acute lymphoblastic leukemia; HR: hazard ratio; AML: acute myeloid leukemia; CNS: central nervous system; UFP: ultrafine particle concentrations; BC: black carbon; SIA: secondary inorganic aerosols; NHL: non-Hodgkin’s lymphoma; PM10: particulate matter smaller than 10 microns; GCT: germ cell tumors; SNST: sympathetic nervous system tumor; PAH: polycyclic aromatic hydrocarbons; ANLL: acute non-lymphoblastic leukemia; RR: relative risk; CBT: childhood brain tumor; HCS: hydrocarbon solvents; EEF: engine exhaust fumes; ML: myeloid leukemia; HL: Hodgkin’s lymphoma; STS: soft tissue sarcoma; AHS: Agriculture Health Study; PCP: pentachlorophenol; RM: ratio of means; RMS: rhabdomyosarcoma; PCB: polychlorinated biphenyls; PBDES: polybrominated diphenyl ethers; ELF-MF: extremely low-frequency magnetic fields; RMF: residential magnetic fields; HVOL: high-voltage. Colors indicate the degree of associations: positive [green], low [yellow], or no [red] associations.

**Table 5:** Studies assessing exposure to indoor chemicals and childhood cancer risk.

Reference	Pollutant	Cancer	Population	Results OR 95% CI	Type of Study
Rossides, M., 2022 [140]	Parents occupationally exposed to hydrocarbon solvents and engine exhaust fumes	Childhood cancer	Sweden	Maternal exposure was associated with non-Hodgkin’s lymphoma, germ cell tumors, astrocytoma, myeloid leukemia, lymphomas, and epithelial tumors. Paternal exposure associated with Hodgkin’s lymphoma.	Case–control: 9653 cases of maternal and 12,521 cases of paternal exposure
Zhang, 2021 [141]	Indoor volatile organic compounds exposures	Leukemia	Shanghai	Styrene 2.33, 1.07–5.07, and butyl alcohol 2.51, 1.19–5.28 with increased risk of AL	Case–control: 97 cases
Stayner, 2021 [142]	Exposure to nitrate from drinking water	Childhood cancer	Denmark	CNC and the highest category of nitrate exposure [>25 mg/L nitrate] was observed for preconception 1.82, 1.09–3.04, prenatal 1.65, 0.97–2.81, and postnatal exposure 1.48, 0.82–2.68.	Case–control: 596 cases
Hvidtfeldt, 2020 [143]	Air pollution exposure at the residence	Childhood cancer	Denmark	All cancers combined 0·97, 0·94–1·01 per 10 µg/m^3^ NO_2_, 0·89, 0·82–0·98 per 5 µg/m^3^ PM2.5, and 0·94, 0·88–1·01 per 1 µg/m^3^ BC. AP exposure and NHL 1·21, 0·94–1·55 per 10 µg/m3 NO_2_, 2·11, 1·10–4·01 per 5 µg/m^3^ PM2.5, and 1·68, 1·06–2·66 per 1 µg/m^3^ BC	Case–control: 5045 cases
Wang, 2019 [144]	Maternal prenatal exposure to pesticides and several environmental factors	ALL	China	Maternal prenatal exposure to interior housing renovation 2.98, 1.51–5.86 or pesticides 1.48, 1.67–2.28 and increased the risk of ALL.	Case–control: 345 cases
Raaschou-Nielsen, 2018 [145]	Ambient benzene at the residence	Leukemia, lymphoma, and CNS tumor	Denmark	Benzene and ALL, and AML 1.0, 0.6–1.7 and 1.9, 0.3–11.1	Case–control: 1989 cases
Ghosh, S., 2018 [146]	Polychlorinated biphenyls	Childhood cancer	Slovakia	PCB exposures, even at the early age of these children, may have lifelong consequences for the future development of chronic diseases.	Cohort: 175 children
Eerjaee, 2017 [92]	Diverse environmental factors	Childhood cancers	Iran	Maternal oral contraceptive pill uses during pregnancy 1.85, 0.67–5.15; radiation during pregnancy 2.15, 0.74–4.61; parental smoking 1.56, 0.73–3.36; residence near high-voltage electricity lines 2.12, 1.17–3.84; pesticides and fertilizers 2.27, 1.26–4.08; patient allergy 2.09, 1.04–4.22; contact with domestic animals 2.24, 1.43–3.50 associated with cancer	Case–control: 300 cases
Park, S., 2017 [147]	Exposure to ambient dichloromethane in pregnancy	Childhood cancer	California	Dichloromethane in pregnancy and germ cell tumors 1.52, 1.11–2.08, particularly teratomas 2.08, 1.38–3.13, and possible increased risk for AML 1.64, 1.15–2.32	Case–control: 13,636 cases
Whitehead, P., 2017 [148]	Home remodeling	Leukemia		Construction in the home between birth and diagnosis associated with ALL 1.52, 1.14–2.02	Case- control. 609 ALL and 89 AML
Metayer, C., 2016 [149]	Paretnal occupational exposure to organic solvents	Leukemia	California	Exposure to any organic solvents in Latino fathers and ALL 1.48, 1.01–2.16. In multivariable analyses, for chlorinated hydrocarbons was 2.28, 0.97–5.37. Combustion exhaust/polycyclic aromatic hydrocarbons [PAHs] and ALL 1.70, 1.16–2.57, and 1.46, 0.94–2.26 with and without adjustment for chlorinated hydrocarbons, respectively.	Case–control: 774 cases
Jiang, W.C., 2016 [150]	Paint, maternal chemical exposure during pregnancy, paternal diesel or gasoline exposure, paternal dye exposure, trash burning near the child's residence, benzene, and formaldehyde	ALL	China	Benzene exposure 1.09, 1.00–1.19, home painting in the past 10 years 3.56, 1.20–10.53, and paternal diesel or gasoline exposure 3.75, 1.06–13.22 were associated with increased risk of cALL.	Case–control: 71 cases
Bailey, D., 2015 [151]	Home paint exposures	Leukemia	France	Home paint exposure in the 1–3 months before conception and risk of ALL was 1.54, 1.28–1.85; for exposure in the year before conception, it was 1.00, 0.86–1.17. For exposure during pregnancy, was 1.14, 1.04–1.25, and for exposure after birth, was 1.22, 1.07–1.39.	Case–control: 3002 cases
Ward, H., 2014 [152]	Residential levels of polybrominated diphenyl ethers [PBDEs]	ALL	California	Homes in the highest PBDES concentration [nanograms per gram] and ALL risk. For PBDE-196 2.1, 1.1–3.8, PBDE-203 2.0, 1.1–3.6; PBDE-206 2.1, 1.1–3.9, and PBDE-207 2.0, 1.03–3.8	Case–control: 167 cases
Gao Yu, 2014 [153]	Indoor air pollution	Leukemia	Shanghai	Higher concentrations of NO_2_ 5.87, 2.25–15.30, benzene 2.56, 1.04–6.28, toluene 2.46, 1.02–5.93, styrene 4.39, 1.90–10.17, chloroform 30.00, 4.09–219.99, butyl alcohol 2.19, 1.03–4.66, methyl ethyl ketone 3.89, 1.55–9.78, and methyl isobutyl ketone 5.32, 1.61–17.58 were related to ALL	Case–control: 105 cases
Parodi, S., 2014 [154]	Perinatal exposures	Neuroblastoma	Italy	Exposure in pregnancy to chemical products for domestic work and to hair dye; the latter was higher among 0–17-month-old children 5.5, 1.0–29.3 and neuroblastoma. Mothers with exposure in the preconception period to solvents 2.0, 1.0–4.1, particularly to aromatic hydrocarbons 9.2, 2.4–34.3. A higher risk was found among children with congenital malformations 4.9, 1.8–13.6.	Case–control: 153 cases
Peters, S., 2014 [155]	Benzene, other aromatics, aliphatics, and chlorinated solvents in key time periods relative to the birth of their child	Brain tumors	Australia	Increased risk of CBT with maternal occupational exposures to chlorinated solvents 8.59, 0.94–78.9. Paternal exposure to solvents in the year before conception and CBT risk 1.55, 0.99–2.43. This increased risk appeared to be mainly attributable to exposure to aromatic solvents 2.72, 0.94–7.86, for benzene and 1.76, 1.10–2.82 for other aromatics compounds.	Case–control: 306 cases
Ruckart, P.Z., 2013 [156]	Water contaminated with trichloroethylene, tetrachloroethylene, benzene, vinyl chloride, and trans-1,2-dichloroethylene	Hematopoietic cancers	North Carolina, USA	Tetrachloroethylene exposure and any vinyl chloride exposure and hematopoietic cancers 1.69, 0.5–4.8, and 1.6, 0.5–4.7, respectively.	Case–control: 106 cases
Shi, 2013 [157]	Parental exposure to chemicals	Leukemia	China	Maternal exposure to total chemicals [diesel oil, gasoline, paints, insecticides, pesticides, herbicides, and chemical fertilizers] from 3 months before pregnancy to the end of pregnancy 2.9, 1.1–7.8, paternal exposure to insecticides 10.1, 1.2–82.9, and chemical fertilizers 9.5, 1.1–79.6 and leukemia	Case–control: 201 cases

OR 95% CI: odds ratio 95% confidence interval; PM2.5: particulate matter smaller than 2.5 microns; ALL: acute lymphoblastic leukemia; HR: hazard ratio; AML: acute myeloid leukemia; CNS: central nervous system; UFP: ultrafine particle concentrations; BC: black carbon; SIA: secondary inorganic aerosols; NHL: non-Hodgkin’s lymphoma; PM10: particulate matter smaller than 10 microns; GCT: germ cell tumors; SNST: sympathetic nervous system tumor; PAH: polycyclic aromatic hydrocarbons; ANLL: acute non-lymphoblastic leukemia; RR: relative risk; CBT: childhood brain tumor; HCS: hydrocarbon solvents; EEF: engine exhaust fumes; ML: myeloid leukemia; HL: Hodgkin’s lymphoma; STS: soft tissue sarcoma; AHS: Agriculture Health Study; PCP: pentachlorophenol; RM: ratio of means; RMS: rhabdomyosarcoma; PCB: polychlorinated biphenyls; PBDES: polybrominated diphenyl ethers; ELF-MF: extremely low-frequency magnetic fields; RMF: residential magnetic fields; HVOL: high-voltage. Colors indicate the degree of associations: positive [green], low [yellow], or no [red] associations.

**Table 6:** Studies assessing exposure to electromagnetic fields and childhood cancer risk.

Reference	Pollutant	Cancer	Population	Results OR 95% CI	Type of Study
Brabant, C., 2022 [158]	Magnetic fields	Leukemia		Leukemia and ELF-MF 1.26, 1.06–1.49. Magnetic flux density threshold associated with leukemia. Living within 50 m and 200 m of power lines were 1.11, 0.81–1.52 and 0.98, 0.85–1.12, respectively. Living within 50 m of power lines and ALL analyzed separately was 1.44, 0.72–2.88. Finally, the risk of leukemia was increased after exposure to electric blankets 2.75, 1.71–4.42 and, to a lesser extent, electric clocks 1.27, 1.01–1.60.	Systematic review meta-analysis
Amoon, A., 2022 [159]	Magnetic fields	Leukemia		No increased risk of leukemia among children exposed to greater MF	Pooled analysis
Seoumun, G., 2021 [160]	Extremely low-frequency magnetic fields	Childhood cancer		0.2, 0.3, and 0.4 μT ELF-MFs had a 1.26, 1.06–1.49, 1.22, 0.93–1.61, and 1.72, 1.25–2.35-times higher odds of leukemia. In brain tumors, children exposed to 0.2 μT had a 0.95, 0.59–1.56-times higher odds, and those exposed to 0.4 μT ELF-MFs had a 1.25, 0.93–1.61. Children exposed to 0.2 and 0.4 μT ELF-MFs had a 1.10, 0.70–1.75 and 2.01, 0.89–4.52-times higher odds of any cancer.	Systematic review meta-analysis
Núñez-Enríquez, 2020 [161]	Extremely low-frequency magnetic fields	ALL	Mexico	ELF-MF exposure as a continuous variable [per 0.2 μT intervals] was associated with B-ALL risk 1.06, 1.01–1.12.	Case–control: 290 cases
Crespi, C., 2019 [162]	Magnetic fields from power lines	Leukemia	California	No close proximity to high-voltage lines alone nor exposure to high calculated fields alone were associated with leukemia. Group that was both very close to high-voltage lines [<50 m] and had high calculated fields [≥0.4 μT] 4.06, 1.16–14.3.	Case–control
Auger, N., 2019 [163]	Residential exposure to electromagnetic fields	Childhood cancer	Canada	Residential proximity to transformer stations associated with cancer. Compared with 200 m, a distance of 80 m from a transformer station was associated with a hazard ratio of 1.08, 0.98–1.20 for any cancer, 1.04, 0.88–1.23 for hematopoietic cancer, and 1.11, 0.99–1.25 for solid tumors.	Cohort: 1114 cases
Talibov, M., 2019 [164]	Parental occupaitonal exposure to low-frequency magnetic fields	Leukemia		Did not find any associations between parental occupational ELF-MF exposure and leukemia.	Meta-analysis
Amoon, A., 2018 [165]	Proximity to overhead power lines	Leukemia		No association between leukemia and distance to nearest overhead power line of any voltage. Among children living < 50 m from 200+ kV power lines, for leukemia observed 1.33, 0.92–1.93.	Pooled analysis
Su, L., 2018 [166]	Parental occupational exposure to extremely low-frequency magnetic fields	CNS		Parental occupational ELF-MF exposure and increased risk of CNS tumors 1.11, 1.02–1.21. Increased risk of CNS tumors was associated with maternal 1.16, 1.06–1.26 but not paternal 1.15, 0.98–1.34 occupational ELF-MF exposure.	Meta-analysis
Kheifets, L., 2017 [167]	Residential magnetic fields	Leukemia	California	Slight risk deficit in two intermediate exposure groups of RMF and a small excess risk in the highest exposure group 1.50, 0.70–3.23, associated with leukemia	Population-based case–control: 5788 cases
Su, L., 2016 [168]	Parental occupational exposure to extremely low-frequency magnetic fields	Leukemia		No maternal or paternal occupational exposure was associated with leukemia.	Meta-analysis
Crespi, C., 2016 [169]	Distance from power lines	Leukemia	California	For leukemia, there was a slight excess of cases within 50 m of a transmission line over 200 kV, 1.4, 0.7–2.7.	Population-based case–control: 5788 cases
Bunch, K.J., 2016 [170]	High-voltage power lines, electromagnetic fields	Leukemia, CNS	UK	Elevated risks for childhood leukemia and overhead power lines may be higher for older age at diagnosis.	
Dechent, D., 2016 [171]	Electromagnetic fields	Leukemia	Iran	Exposure to high-voltage power lines 3.651, 1.692–7.878 and leukemia.	Case–control
Tabrizi, 2015 [172]	Electromagnetic field exposure	ALL	Tehran, Iran	Exposure to high-voltage power lines 3.651, 1.692–7.878 and ALL.	Case–control: 22 cases
Bunch, J., 2015 [173]	High-voltage underground cables	Childhood cancer	England	No indications of an association of risk with distance or of trend in risk with increasing magnetic field for leukemia, and no convincing pattern of risks for any other cancer.	Case–control: 52,525 cases
Pedersen, C., 2015 [174]	Extremely low-frequency magnetic fields	Childhood leukemia, CNS tumor, and lymphoma	Denmark	RR was 0.88, 0.32–2.42, and, for the total period [1968–2003], it was 1.63, 0.77–3.46 for leukemia, CNS tumour, and malignant lymphoma combined for exposures ≥0.4 μT compared with <0.1 μT.	Case–control: 3277 cases
Salvan, A., 2015 [175]	50 Hz magnetic fields	Leukemia	Italy	Our results may be affected by several sources of bias, and they are noninformative at exposure levels >0.3 μT.	Case–control: 412 cases
Bunch, K.J., 2014 [176]	Distance at birth from overhead high-voltage powerlines	Childhood cancer	Britain	RR for leukemia, 0–199 m compared with >1000 m, all voltages: 1960s 4.50, 0.97–20.83, 2000s 0.71, 0.49–1.03.	Case–control: 53,515 children
Zhao, L., 2013 [177]	Magnetic fields	Leukemia		Positive association between magnetic field intensity ≥0.2 μT and leukemia 1.31, 1.06–1.61. For total leukemia: 1.57, 1.03–2.40; for ALL 2.43, 1.30–4.55.	11,699 cases
Pedersen, C., 2014 [178]	Distance from residence to power line	Leukemia	Denmark	Children living 0–199 m of a power line and being exposed to domestic radon >42 Bq/m^3^ and leukemia RR 2.88, 1.01–8.27. Children living 200–599 m from a power line and being exposed to domestic radon <42 Bq/m^3^ had a lower risk, 0.24, 0.07–0.83	Case–control: 1698 cases
Sermage-Faure, C., 2013 [179]	High-voltage power lines	Leukemia	France	Living within 50 m of a VHV-HVOL and AL 1.7, 0.9–3.6	Case–control: 2779 cases

OR 95% CI: odds ratio 95% confidence interval; PM2.5: particulate matter smaller than 2.5 microns; ALL: acute lymphoblastic leukemia; HR: hazard ratio; AML: acute myeloid leukemia; CNS: central nervous system; UFP: ultrafine particle concentrations; BC: black carbon; SIA: secondary inorganic aerosols; NHL: non-Hodgkin’s lymphoma; PM10: particulate matter smaller than 10 microns; GCT: germ cell tumors; SNST: sympathetic nervous system tumor; PAH: polycyclic aromatic hydrocarbons; ANLL: acute non-lymphoblastic leukemia; RR: relative risk; CBT: childhood brain tumor; HCS: hydrocarbon solvents; EEF: engine exhaust fumes; ML: myeloid leukemia; HL: Hodgkin’s lymphoma; STS: soft tissue sarcoma; AHS: Agriculture Health Study; PCP: pentachlorophenol; RM: ratio of means; RMS: rhabdomyosarcoma; PCB: polychlorinated biphenyls; PBDES: polybrominated diphenyl ethers; ELF-MF: extremely low-frequency magnetic fields; RMF: residential magnetic fields; HVOL: high-voltage. Colors indicate the degree of associations: positive [green], low [yellow], or no [red] associations.

**Table 7:** Studies assessing indoor radon exposure and childhood cancer risk.

Reference	Pollutant	Cancer	Population	Results OR 95% CI	Type of Study
Ngoc, 2022 [180]	Radon	Leukemia	Europe	Pooled OR 1.43, 1.19–1.72	Systematic review and meta-analysis
Moon, J., 2021 [181]	Residential radon exposure	Leukemia		For case–control studies, pooled OR 1.0308, 1.0050–1.0573 increase for each 100 Bq/m^3^ of radon dose. The pooled OR 1.0361, 1.0014–1.0720 increase for each 100 Bq/m^3^ of radon dose for lymphoid leukemia subgroup.	Meta-analysis
Lu, Y., 2020 [182]	Domestic radon exposure	Leukemia		Radon exposure and leukemia 1.22, 1.01–1.42. Cohort studies HR 0.97, 0.81–1.1.	Meta-analysis
Nikkilä, A., 2019 [183]	Residential radon concentration	Leukemia	Finland	Radon and leukemia, second quartile 1.08, 0.77–1.50, third quartile 1.10, 0.79–1.53, and 1.29, 0.93–1.77 for the highest quartile.	Case–control
Chen, J., 2019 [184]	Domestic radon exposure	Leukemia and lymphoma	Canada	AML incidence in group of 5–9 years male with increased radon exposure R2 = 0.53, *p* = 0.026. For females, NHL incidence rates and average radon concentrations occurred in the 5–9 years age group R2 = 0.387, *p* = 0.041.	Population-based registry
Peckham, E., 2015 [185]	Residential radon exposure	Lymphoma	Texas	No evidence that residential radon exposure was positively associated with lymphoma overall, HL, or BL. Areas with radon concentrations >75th percentile had a marginal increase in DLBCL incidence [aIRR = 1.73, 95% CI: 1.03–2.91].	Population-based: 2147 cases
Kollerud, R., 2014 [186]	High indoor radon concentrations	Leukemia and CNS tumors	Norway	Small increased risk of both leukemia and CNS among children under 1 year of age in the highest radon exposure group 1.26, 1.05–1.52, and similar for CNS alone 1.34, 1.04–1.73	Cohort: 864 cases
Hauri, D., 2013 [187]	Domestic radon exposure	Childhood cancer	Switzerland	They did not find evidence that domestic radon exposure is associated with childhood cancer	Prospective census-based cohort: 997 cases

OR 95% CI: odds ratio 95% confidence interval; PM2.5: particulate matter smaller than 2.5 microns; ALL: acute lymphoblastic leukemia; HR: hazard ratio; AML: acute myeloid leukemia; CNS: central nervous system; UFP: ultrafine particle concentrations; BC: black carbon; SIA: secondary inorganic aerosols; NHL: non-Hodgkin’s lymphoma; PM10: particulate matter smaller than 10 microns; GCT: germ cell tumors; SNST: sympathetic nervous system tumor; PAH: polycyclic aromatic hydrocarbons; ANLL: acute non-lymphoblastic leukemia; RR: relative risk; CBT: childhood brain tumor; HCS: hydrocarbon solvents; EEF: engine exhaust fumes; ML: myeloid leukemia; HL: Hodgkin’s lymphoma; STS: soft tissue sarcoma; AHS: Agriculture Health Study; PCP: pentachlorophenol; RM: ratio of means; RMS: rhabdomyosarcoma; PCB: polychlorinated biphenyls; PBDES: polybrominated diphenyl ethers; ELF-MF: extremely low-frequency magnetic fields; RMF: residential magnetic fields; HVOL: high-voltage. Colors indicate the degree of associations: positive [green], low [yellow], or no [red] associations.

## References

[1] Manisalidis I., Stavropoulou E., Stavropoulos A., Bezirtzoglou E. (2020). Environmental and Health Impacts of Air Pollution: A Review. Front. Public Health.

[2] Buser J.M., Lake K., Ginier E. (2022). Environmental Risk Factors for Childhood Cancer in an Era of Global Climate Change: A Scoping Review. J. Pediatr. Health Care Off. Publ. Natl. Assoc. Pediatr. Nurse Assoc. Pract..

[3] Cazzolla Gatti R. (2021). Why We Will Continue to Lose Our Battle with Cancers If We Do Not Stop Their Triggers from Environmental Pollution. Int. J. Environ. Res. Public Health.

[4] Childhood Cancer Inequalities in the WHO European Region. https://www.who.int/europe/publications/i/item/9789289057615.

[5] PAHO WHO|Pan American Health Organization Childhood and Adolescence Cancer. https://www.paho.org/en/topics/childhood-and-adolescence-cancer.

[6] Siegel R.L., Miller K.D., Wagle N.S., Jemal A. (2023). Cancer statistics, 2023. CA Cancer J. Clin..

[7] Fucic A., Guszak V., Mantovani A. (2017). Transplacental exposure to environmental carcinogens: Association with childhood cancer risks and the role of modulating factors. Reprod. Toxicol..

[8] Schmidt J.A., Hornhardt S., Erdmann F., Sánchez-García I., Fischer U., Schüz J., Ziegelberger G. (2021). Risk Factors for Childhood Leukemia: Radiation and Beyond. Front. Public Health.

[9] Greaves M. (2018). A causal mechanism for childhood acute lymphoblastic leukaemia. Nat. Rev. Cancer.

[10] Risk Factors and Causes of Childhood Cancer. https://www.cancer.org/cancer/cancer-in-children/risk-factors-and-causes.html.

[11] Iqbal S., Ali S., Ali I. (2022). Maternal pesticide exposure and its relation to childhood cancer: An umbrella review of meta-analyses. Int. J. Environ. Health Res..

[12] Zhong C., Wang R., Morimoto L.M., Longcore T., Franklin M., Rogne T., Metayer C., Wiemels J.L., Ma X. (2023). Outdoor artificial light at night, air pollution, and risk of childhood acute lymphoblastic leukemia in the California Linkage Study of Early-Onset Cancers. Sci. Rep..

[13] Malavolti M., Malagoli C., Filippini T., Wise L.A., Bellelli A., Palazzi G., Cellini M., Costanzini S., Teggi S., Vinceti M. (2023). Residential proximity to petrol stations and risk of childhood leukemia. Eur. J. Epidemiol..

[14] Kreis C., Héritier H., Scheinemann K., Hengartner H., de Hoogh K., Röösli M., Spycher B.D. (2022). Childhood cancer and traffic-related air pollution in Switzerland: A nationwide census-based cohort study. Environ. Int..

[15] Lee J.M., Lee T.H., Kim S., Song M., Bae S. (2022). Association between long-term exposure to particulate matter and childhood cancer: A retrospective cohort study. Environ. Res..

[16] Mazzei A., Konstantinoudis G., Kreis C., Diezi M., Ammann R.A., Zwahlen M., Kühni C., Spycher B.D. (2022). Childhood cancer and residential proximity to petrol stations: A nationwide registry-based case-control study in Switzerland and an updated meta-analysis. Int. Arch. Occup. Environ. Health.

[17] Asenjo S., Nuñez O., Segú-Tell J., Pardo Romaguera E., Cañete Nieto A., Martín-Méndez I., Bel-Lan A., García-Pérez J., Cárceles-Álvarez A., Ortega-García J.A. (2022). Cadmium [Cd] and Lead [Pb] topsoil levels and incidence of childhood leukemias. Environ. Geochem. Health.

[18] Onyije F.M., Hosseini B., Togawa K., Schüz J., Olsson A. (2021). Cancer Incidence and Mortality among Petroleum Industry Workers and Residents Living in Oil Producing Communities: A Systematic Review and Meta-Analysis. Int. J. Environ. Res. Public Health.

[19] Ribeiro A.G., Vermeulen R., Cardoso M.R.A., de Oliveira Latorre M.d.R.D., Hystad P., Downward G.S., Nardocci A.C. (2021). Residential traffic exposure and lymphohematopoietic malignancies among children in the city of São Paulo, Brazil: An ecological study. Cancer Epidemiol..

[20] Lavigne E., Lima I., Hatzopoulou M., Van Ryswyk K., van Donkelaar A., Martin R.V., Chen H., Stieb D.M., Crighton E., Burnett R.T. (2020). Ambient ultrafine particle concentrations and incidence of childhood cancers. Environ. Int..

[21] Hvidtfeldt U.A., Erdmann F., Urhoj S.K., Brandt J., Geels C., Ketzel M., Frohn L.M., Christensen J.H., Sørensen M., Raaschou-Nielsen O. (2020). Residential Exposure to PM2.5 Components and Risk of Childhood Non-Hodgkin Lymphoma in Denmark: A Nationwide Register-Based Case-Control Study. Int. J. Environ. Res. Public Health.

[22] Volk J., Heck J.E., Schmiegelow K., Hansen J. (2019). Parental occupational exposure to diesel engine exhaust in relation to childhood leukaemia and central nervous system cancers: A register-based nested case-control study in Denmark 1968–2016. Occup. Environ. Med..

[23] Peckham-Gregory E.C., Ton M., Rabin K.R., Danysh H.E., Scheurer M.E., Lupo P.J. (2019). Maternal Residential Proximity to Major Roadways and the Risk of Childhood Acute Leukemia: A Population-Based Case-Control Study in Texas, 1995–2011. Int. J. Environ. Res. Public Health.

[24] García-Pérez J., Gómez-Barroso D., Tamayo-Uria I., Ramis R. (2019). Methodological approaches to the study of cancer risk in the vicinity of pollution sources: The experience of a population-based case-control study of childhood cancer. Int. J. Health Geogr..

[25] Gong Z.H., Li J., Wang X.Y., Yu Y., Ren M.M., Zhou J. (2019). A Meta-analysis of Traffic-related Air Pollution and Risk of Childhood Leukemia. J. Pediatr. Hematol. Oncol..

[26] Filippini T., Hatch E.E., Rothman K.J., Heck J.E., Park A.S., Crippa A., Orsini N., Vinceti M. (2019). Association between Outdoor Air Pollution and Childhood Leukemia: A Systematic Review and Dose-Response Meta-Analysis. Environ. Health Perspect..

[27] Seifi M., Niazi S., Johnson G., Nodehi V., Yunesian M. (2019). Exposure to ambient air pollution and risk of childhood cancers: A population-based study in Tehran, Iran. Sci. Total Environ..

[28] Hall C., Heck J.E., Ritz B., Cockburn M., Escobedo L.A., von Ehrenstein O.S. (2019). Prenatal Exposure to Air Toxics and Malignant Germ Cell Tumors in Young Children. J. Occup. Environ. Med..

[29] Iavarone I., Buzzoni C., Stoppa G., Steliarova-Foucher E., SENTIERI-AIRTUM Working Group (2018). Cancer incidence in children and young adults living in industrially contaminated sites: From the Italian experience to the development of an international surveillance system. Epidemiol. Prev..

[30] Kirkeleit J., Riise T., Bjørge T., Christiani D.C., Bråtveit M., Baccarelli A., Mattioli S., Hollund B.E., Gjertsen B.T. (2018). Maternal exposure to gasoline and exhaust increases the risk of childhood leukaemia in offspring—A prospective study in the Norwegian Mother and Child Cohort Study. Br. J. Cancer.

[31] Kumar S.V., Lupo P.J., Pompeii L.A., Danysh H.E. (2018). Maternal Residential Proximity to Major Roadways and Pediatric Embryonal Tumors in Offspring. Int. J. Environ. Res. Public Health.

[32] Ortega-García J.A., López-Hernández F.A., Cárceles-Álvarez A., Fuster-Soler J.L., Sotomayor D.I., Ramis R. (2017). Childhood cancer in small geographical areas and proximity to air-polluting industries. Environ. Res..

[33] Janitz A.E., Campbell J.E., Magzamen S., Pate A., Stoner J.A., Peck J.D. (2017). Benzene and childhood acute leukemia in Oklahoma. Environ. Res..

[34] Ramis R., Tamayo-Uria I., Gómez-Barroso D., López-Abente G., Morales-Piga A., Pardo Romaguera E., Aragones N., García-Pérez J. (2017). Risk factors for central nervous system tumors in children: New findings from a case-control study. PLoS ONE.

[35] Lavigne É., Bélair M.A., Do M.T., Stieb D.M., Hystad P., van Donkelaar A., Martin R.V., Crouse D.L., Crighton E., Chen H. (2017). Maternal exposure to ambient air pollution and risk of early childhood cancers: A population-based study in Ontario, Canada. Environ. Int..

[36] Spycher B.D., Lupatsch J.E., Huss A., Rischewski J., Schindera C., Spoerri A., Vermeulen R., Kuehni C.E., Swiss Paediatric Oncology Group, Swiss National Cohort Study Group (2017). Parental occupational exposure to benzene and the risk of childhood cancer: A census-based cohort study. Environ. Int..

[37] García-Pérez J., Morales-Piga A., Gómez-Barroso D., Tamayo-Uria I., Pardo Romaguera E., López-Abente G., Ramis R. (2017). Risk of bone tumors in children and residential proximity to industrial and urban areas: New findings from a case-control study. Sci. Total Environ..

[38] Janitz A.E., Ramachandran G., Tomlinson G.E., Krailo M., Richardson M., Spector L. (2017). Maternal and paternal occupational exposures and hepatoblastoma: Results from the HOPE study through the Children’s Oncology Group. J. Expo. Sci. Environ. Epidemiol..

[39] Janitz A.E., Campbell J.E., Magzamen S., Pate A., Stoner J.A., Peck J.D. (2016). Traffic-related air pollution and childhood acute leukemia in Oklahoma. Environ. Res..

[40] Danysh H.E., Zhang K., Mitchell L.E., Scheurer M.E., Lupo P.J. (2016). Maternal residential proximity to major roadways at delivery and childhood central nervous system tumors. Environ. Res..

[41] von Ehrenstein O.S., Heck J.E., Park A.S., Cockburn M., Escobedo L., Ritz B. (2016). In Utero and Early-Life Exposure to Ambient Air Toxics and Childhood Brain Tumors: A Population-Based Case-Control Study in California, USA. Environ. Health Perspect..

[42] Symanski E., Tee Lewis P.G., Chen T.Y., Chan W., Lai D., Ma X. (2016). Air toxics and early childhood acute lymphocytic leukemia in Texas, a population based case control study. Environ. Health Glob. Access Sci. Source.

[43] Magnani C., Ranucci A., Badaloni C., Cesaroni G., Ferrante D., Miligi L., Mattioli S., Rondelli R., Bisanti L., Zambon P. (2016). Road Traffic Pollution and Childhood Leukemia: A Nationwide Case-control Study in Italy. Arch. Med. Res..

[44] García-Pérez J., Morales-Piga A., Gómez J., Gómez-Barroso D., Tamayo-Uria I., Romaguera E.P., Fernández-Navarro P., López-Abente G., Ramis R. (2016). Association between residential proximity to environmental pollution sources and childhood renal tumors. Environ. Res..

[45] García-Pérez J., Morales-Piga A., Gómez-Barroso D., Tamayo-Uria I., Pardo Romaguera E., López-Abente G., Ramis R. (2016). Residential proximity to environmental pollution sources and risk of rare tumors in children. Environ. Res..

[46] García-Pérez J., Morales-Piga A., Gómez-Barroso D., Tamayo-Uria I., Pardo Romaguera E., Fernández-Navarro P., Lopez-Abente G., Ramis R. (2016). Risk of neuroblastoma and residential proximity to industrial and urban sites: A case-control study. Environ. Int..

[47] Carlos-Wallace F.M., Zhang L., Smith M.T., Rader G., Steinmaus C. (2016). Parental, In Utero, and Early-Life Exposure to Benzene and the Risk of Childhood Leukemia: A Meta-Analysis. Am. J. Epidemiol..

[48] Filippini T., Heck J.E., Malagoli C., Del Giovane C., Vinceti M. (2015). A review and meta-analysis of outdoor air pollution and risk of childhood leukemia. J. Environ. Sci. Health Part C Environ. Carcinog. Ecotoxicol. Rev..

[49] Spycher B.D., Feller M., Röösli M., Ammann R.A., Diezi M., Egger M., Kuehni C.E. (2015). Childhood cancer and residential exposure to highways: A nationwide cohort study. Eur. J. Epidemiol..

[50] Malagoli C., Malavolti M., Costanzini S., Fabbri S., Tezzi S., Palazzi G., Arcolin E., Vinceti M. (2015). Increased incidence of childhood leukemia in urban areas: A population-based case-control study. Epidemiol. Prev..

[51] Heck J.E., Park A.S., Qiu J., Cockburn M., Ritz B. (2015). Retinoblastoma and ambient exposure to air toxics in the perinatal period. J. Expo. Sci. Environ. Epidemiol..

[52] Houot J., Marquant F., Goujon S., Faure L., Honoré C., Roth M.H., Hémon D., Clavel J. (2015). Residential Proximity to Heavy-Traffic Roads, Benzene Exposure, and Childhood Leukemia-The GEOCAP Study, 2002–2007. Am. J. Epidemiol..

[53] Greenop K.R., Hinwood A.L., Fritschi L., Scott R.J., Attia J., Ashton L.J., Heath J.A., Armstrong B.K., Milne E. (2015). Vehicle refuelling, use of domestic wood heaters and the risk of childhood brain tumours: Results from an Australian case-control study. Pediatr. Blood Cancer.

[54] García-Pérez J., López-Abente G., Gómez-Barroso D., Morales-Piga A., Romaguera E.P., Tamayo I., Fernández-Navarro P., Ramis R. (2015). Childhood leukemia and residential proximity to industrial and urban sites. Environ. Res..

[55] Zhou Y., Zhang S., Li Z., Zhu J., Bi Y., Bai Y., Wang H. (2014). Maternal benzene exposure during pregnancy and risk of childhood acute lymphoblastic leukemia: A meta-analysis of epidemiologic studies. PLoS ONE.

[56] Shrestha A., Ritz B., Wilhelm M., Qiu J., Cockburn M., Heck J.E. (2014). Prenatal exposure to air toxics and risk of Wilms tumor in 0- to 5-year-old children. J. Occup. Environ. Med..

[57] Boothe V.L., Boehmer T.K., Wendel A.M., Yip F.Y. (2014). Residential traffic exposure and childhood leukemia: A systematic review and meta-analysis. Am. J. Prev. Med..

[58] Heck J.E., Park A.S., Qiu J., Cockburn M., Ritz B. (2014). Risk of leukemia in relation to exposure to ambient air toxics in pregnancy and early childhood. Int. J. Hyg. Environ. Health.

[59] Badaloni C., Ranucci A., Cesaroni G., Zanini G., Vienneau D., Al-Aidrous F., De Hoogh K., Magnani C., Forastiere F., SETIL Study Group (2013). Air pollution and childhood leukaemia: A nationwide case-control study in Italy. Occup. Environ. Med..

[60] Heck J.E., Wu J., Lombardi C., Qiu J., Meyers T.J., Wilhelm M., Cockburn M., Ritz B. (2013). Childhood cancer and traffic-related air pollution exposure in pregnancy and early life. Environ. Health Perspect..

[61] Ghosh J.K.C., Heck J.E., Cockburn M., Su J., Jerrett M., Ritz B. (2013). Prenatal exposure to traffic-related air pollution and risk of early childhood cancers. Am. J. Epidemiol..

[62] Peters S., Glass D.C., Reid A., de Klerk N., Armstrong B.K., Kellie S., Ashton L.J., Milne E., Fritschi L. (2013). Parental occupational exposure to engine exhausts and childhood brain tumors. Int. J. Cancer.

[63] Heck J.E., Park A.S., Qiu J., Cockburn M., Ritz B. (2013). An exploratory study of ambient air toxics exposure in pregnancy and the risk of neuroblastoma in offspring. Environ. Res..

[64] Ward M.H., Madrigal J.M., Jones R.R., Friesen M.C., Falk R.T., Koebel D., Metayer C. (2023). Glyphosate in house dust and risk of childhood acute lymphoblastic leukemia in California. Environ. Int..

[65] Rafeeinia A., Asadikaram G., Moazed V., Darabi M.K. (2023). Organochlorine pesticides may induce leukemia by methylation of CDKN2B and MGMT promoters and histone modifications. Gene.

[66] Rossides M., Kampitsi C.E., Talbäck M., Mogensen H., Wiebert P., Tettamanti G., Feychting M. (2022). Occupational exposure to pesticides in mothers and fathers and risk of cancer in the offspring: A register-based case-control study from Sweden [1960–2015]. Environ. Res..

[67] Thompson S., Ritz B., Cockburn M., Heck J.E. (2022). Prenatal ambient pesticide exposure and childhood retinoblastoma. Int. J. Hyg. Environ. Health.

[68] Khan A., Feulefack J., Sergi C.M. (2022). Pre-conceptional and prenatal exposure to pesticides and pediatric neuroblastoma. A meta-analysis of nine studies. Environ. Toxicol. Pharmacol..

[69] El-Helaly S., Khashaba E., El Domiaty H., Darwish A. (2022). Parental occupational and environmental risk factors for childhood bone cancer in Mansoura oncology center: A case control study. Int. J. Environ. Health Res..

[70] Khan A., Feulefack J., Sergi C.M. (2022). Exposure to pesticides and pediatric Wilms’ tumor. A meta-analysis on pre-conception and pregnancy parental exposure with an IARC/WHO commentary. Hum. Exp. Toxicol..

[71] Bamouni S., Hémon D., Faure L., Clavel J., Goujon S. (2022). Residential proximity to croplands at birth and childhood leukaemia. Environ. Health Glob. Access Sci. Source.

[72] Onyije F.M., Olsson A., Erdmann F., Magnani C., Petridou E., Clavel J., Miligi L., Bonaventure A., Ferrante D., Piro S. (2022). Parental occupational exposure to combustion products, metals, silica and asbestos and risk of childhood leukaemia: Findings from the Childhood Cancer and Leukaemia International Consortium [CLIC]. Environ. Int..

[73] Feulefack J., Khan A., Forastiere F., Sergi C.M. (2021). Parental Pesticide Exposure and Childhood Brain Cancer: A Systematic Review and Meta-Analysis Confirming the IARC/WHO Monographs on Some Organophosphate Insecticides and Herbicides. Children.

[74] Nguyen A., Crespi C.M., Vergara X., Kheifets L. (2022). Commercial outdoor plant nurseries as a confounder for electromagnetic fields and childhood leukemia risk. Environ. Res..

[75] Madrigal J.M., Jones R.R., Gunier R.B., Whitehead T.P., Reynolds P., Metayer C., Ward M.H. (2021). Residential exposure to carbamate, organophosphate, and pyrethroid insecticides in house dust and risk of childhood acute lymphoblastic leukemia. Environ. Res..

[76] Coste A., Bailey H.D., Kartal-Kaess M., Renella R., Berthet A., Spycher B.D. (2020). Parental occupational exposure to pesticides and risk of childhood cancer in Switzerland: A census-based cohort study. BMC Cancer.

[77] Patel D.M., Jones R.R., Booth B.J., Olsson A.C., Kromhout H., Straif K., Vermeulen R., Tikellis G., Paltiel O., Golding J. (2020). Parental occupational exposure to pesticides, animals and organic dust and risk of childhood leukemia and central nervous system tumors: Findings from the International Childhood Cancer Cohort Consortium [I4C]. Int. J. Cancer.

[78] Park A.S., Ritz B., Yu F., Cockburn M., Heck J.E. (2020). Prenatal pesticide exposure and childhood leukemia—A California statewide case-control study. Int. J. Hyg. Environ. Health.

[79] Mavoungou S., Rios P., Pacquement H., Nolla M., Rigaud C., Simonin M., Bertrand Y., Lambilliotte A., Faure L., Orsi L. (2020). Maternal exposure to pesticides and risk of childhood lymphoma in France: A pooled analysis of the ESCALE and ESTELLE studies [SFCE]. Cancer Epidemiol..

[80] Rios P., Bauer H., Schleiermacher G., Pasqualini C., Boulanger C., Thebaud E., Gandemer V., Pellier I., Verschuur A., Sudour-Bonnange H. (2020). Environmental exposures related to parental habits in the perinatal period and the risk of Wilms’ tumor in children. Cancer Epidemiol..

[81] Coste A., Goujon S., Faure L., Hémon D., Clavel J. (2020). Agricultural crop density in the municipalities of France and incidence of childhood leukemia: An ecological study. Environ. Res..

[82] Patel D.M., Gyldenkærne S., Jones R.R., Olsen S.F., Tikellis G., Granström C., Dwyer T., Stayner L.T., Ward M.H. (2020). Residential proximity to agriculture and risk of childhood leukemia and central nervous system tumors in the Danish national birth cohort. Environ. Int..

[83] Van Maele-Fabry G., Gamet-Payrastre L., Lison D. (2019). Household exposure to pesticides and risk of leukemia in children and adolescents: Updated systematic review and meta-analysis. Int. J. Hyg. Environ. Health.

[84] Bunch K.J., Kendall G.M., Stiller C.A., Vincent T.J., Murphy M.F.G. (2019). Case-control study of paternal occupational exposures and childhood lymphoma in Great Britain, 1962–2010. Br. J. Cancer.

[85] Georgakis M.K., Dessypris N., Papadakis V., Tragiannidis A., Bouka E., Hatzipantelis E., Moschovi M., Papakonstantinou E., Polychronopoulou S., Sgouros S. (2019). Perinatal and early life risk factors for childhood brain tumors: Is instrument-assisted delivery associated with higher risk?. Cancer Epidemiol..

[86] Hyland C., Gunier R.B., Metayer C., Bates M.N., Wesseling C., Mora A.M. (2018). Maternal residential pesticide use and risk of childhood leukemia in Costa Rica. Int. J. Cancer.

[87] Ferri G.M., Guastadisegno C.M., Intranuovo G., Cavone D., Birtolo F., Cecinati V., Pappalardi B., Corsi P., Vimercati L., Santoro N. (2018). Maternal Exposure to Pesticides, Paternal Occupation in the Army/Police Force, and CYP2D6*4 Polymorphism in the Etiology of Childhood Acute Leukemia. J. Pediatr. Hematol. Oncol..

[88] Vidart d’Egurbide Bagazgoïtia N., Bailey H.D., Orsi L., Lacour B., Guerrini-Rousseau L., Bertozzi A.I., Leblond P., Faure-Conter C., Pellier I., Freycon C. (2018). Maternal residential pesticide use during pregnancy and risk of malignant childhood brain tumors: A pooled analysis of the ESCALE and ESTELLE studies [SFCE]. Int. J. Cancer.

[89] Boffetta P., Desai V. (2018). Exposure to permethrin and cancer risk: A systematic review. Crit. Rev. Toxicol..

[90] Van Maele-Fabry G., Gamet-Payrastre L., Lison D. (2017). Residential exposure to pesticides as risk factor for childhood and young adult brain tumors: A systematic review and meta-analysis. Environ. Int..

[91] Gunier R.B., Kang A., Hammond S.K., Reinier K., Lea C.S., Chang J.S., Does M., Scelo G., Kirsch J., Crouse V. (2017). A task-based assessment of parental occupational exposure to pesticides and childhood acute lymphoblastic leukemia. Environ. Res..

[92] Eerjaee A., Niknam M., Sadeghi A., Dehghani M., Safaei Z., Teshnizi S.H., Karimi M. (2017). A Significant Breakthrough in the Incidence of Childhood Cancers and Evaluation of its Risk Factors in Southern Iran. Indian J. Med. Paediatr. Oncol. Off. J. Indian Soc. Med. Paediatr. Oncol..

[93] Rios P., Bailey H.D., Lacour B., Valteau-Couanet D., Michon J., Bergeron C., Boutroux H., Defachelles A.S., Gambart M., Sirvent N. (2017). Maternal use of household pesticides during pregnancy and risk of neuroblastoma in offspring. A pooled analysis of the ESTELLE and ESCALE French studies [SFCE]. Cancer Causes Control CCC..

[94] Omidakhsh N., Ganguly A., Bunin G.R., von Ehrenstein O.S., Ritz B., Heck J.E. (2017). Residential Pesticide Exposures in Pregnancy and the Risk of Sporadic Retinoblastoma: A Report From the Children’s Oncology Group. Am. J. Ophthalmol..

[95] Febvey O., Schüz J., Bailey H.D., Clavel J., Lacour B., Orsi L., Lightfoot T., Roman E., Vermeulen R., Kromhout H. (2016). Risk of Central Nervous System Tumors in Children Related to Parental Occupational Pesticide Exposures in three European Case-Control Studies. J. Occup. Environ. Med..

[96] Gómez-Barroso D., García-Pérez J., López-Abente G., Tamayo-Uria I., Morales-Piga A., Pardo Romaguera E., Ramis R. (2016). Agricultural crop exposure and risk of childhood cancer: New findings from a case-control study in Spain. Int. J. Health Geogr..

[97] Malagoli C., Costanzini S., Heck J.E., Malavolti M., De Girolamo G., Oleari P., Palazzi G., Teggi S., Vinceti M. (2016). Passive exposure to agricultural pesticides and risk of childhood leukemia in an Italian community. Int. J. Hyg. Environ. Health.

[98] Chen S., Gu S., Wang Y., Yao Y., Wang G., Jin Y., Wu Y. (2016). Exposure to pyrethroid pesticides and the risk of childhood brain tumors in East China. Environ. Pollut..

[99] Chen M., Chang C.H., Tao L., Lu C. (2015). Residential Exposure to Pesticide During Childhood and Childhood Cancers: A Meta-Analysis. Pediatrics.

[100] Maryam Z., Sajad A., Maral N., Zahra L., Sima P., Zeinab A., Zahra M., Fariba E., Sezaneh H., Davood M. (2015). Relationship between exposure to pesticides and occurrence of acute leukemia in Iran. Asian Pac. J. Cancer Prev. APJCP.

[101] Zhang Y., Gao Y., Shi R., Chen D., Wang X., Kamijima M., Sakai K., Nakajima T., Khalequzzaman M., Zhou Y. (2015). Household pesticide exposure and the risk of childhood acute leukemia in Shanghai, China. Environ. Sci. Pollut. Res. Int..

[102] Chen D., Zhang Y., Tian Y., Shi R., Wang X., Hu Y., Ji X., Han K., Hu S., Mao S. (2015). Relationship between risk of childhood acute leukemia and children’s and parents’ lifestyles and household environment exposure. Chin. J. Prev. Med..

[103] Bailey H.D., Infante-Rivard C., Metayer C., Clavel J., Lightfoot T., Kaatsch P., Roman E., Magnani C., Spector L.G., Th Petridou E.T. (2015). Home pesticide exposures and risk of childhood leukemia: Findings from the childhood leukemia international consortium. Int. J. Cancer.

[104] Zheng R., Zhang Q., Zhang Q., Yang L., Zhang Z., Huang F. (2015). Occupational exposure to pentachlorophenol causing lymphoma and hematopoietic malignancy for two generations. Toxicol. Ind. Health.

[105] Kunkle B., Bae S., Singh K.P., Roy D. (2014). Increased risk of childhood brain tumors among children whose parents had farm-related pesticide exposures during pregnancy. JP J. Biostat..

[106] Kumar A., Vashist M., Rathee R. (2014). Maternal factors and risk of childhood leukemia. Asian Pac. J. Cancer Prev. APJCP.

[107] Bailey H.D., Fritschi L., Infante-Rivard C., Glass D.C., Miligi L., Dockerty J.D., Lightfoot T., Clavel J., Roman E., Spector L.G. (2014). Parental occupational pesticide exposure and the risk of childhood leukemia in the offspring: Findings from the childhood leukemia international consortium. Int. J. Cancer.

[108] Van Maele-Fabry G., Hoet P., Lison D. (2013). Parental occupational exposure to pesticides as risk factor for brain tumors in children and young adults: A systematic review and meta-analysis. Environ. Int..

[109] Ferreira J.D., Couto A.C., Pombo-de-Oliveira M.S., Koifman S., Brazilian Collaborative Study Group of Infant Acute Leukemia (2013). In utero pesticide exposure and leukemia in Brazilian children < 2 years of age. Environ. Health Perspect..

[110] Greenop K.R., Peters S., Bailey H.D., Fritschi L., Attia J., Scott R.J., Glass D.C., De Klerk N.H., Alvaro F., Armstrong B.K. (2013). Exposure to pesticides and the risk of childhood brain tumors. Cancer Causes Control CCC.

[111] Metayer C., Colt J.S., Buffler P.A., Reed H.D., Selvin S., Crouse V., Ward M.H. (2013). Exposure to herbicides in house dust and risk of childhood acute lymphoblastic leukemia. J. Expo. Sci. Environ. Epidemiol..

[112] Abdolahi A., van Wijngaarden E., McClean M.D., Herrick R.F., Allen J.G., Ganguly A., Bunin G.R. (2013). A case-control study of paternal occupational exposures and the risk of childhood sporadic bilateral retinoblastoma. Occup. Environ. Med..

[113] Wimberly C.E., Gulrajani N.B., Russ J.B., Landi D., Wiemels J.L., Towry L., Wiencke J.K., Walsh K.M. (2024). Maternal prenatal use of alcohol, tobacco, and illicit drugs and associations with childhood cancer subtypes. Cancer Epidemiol. Biomark. Prev. Publ. Am. Assoc. Cancer Res. Cosponsored Am. Soc. Prev. Oncol..

[114] Xu K., Li S., Whitehead T.P., Pandey P., Kang A.Y., Morimoto L.M., Kogan S.C., Metayer C., Wiemels J.L., de Smith A.J. (2021). Epigenetic biomarkers of prenatal tobacco smoke exposure are associated with gene deletions in childhood acute lymphoblastic leukemia. Cancer Epidemiol. Biomark. Prev. Publ. Am. Assoc. Cancer Res. Cosponsored Am. Soc. Prev. Oncol..

[115] Alyahya M.S., Al-Sheyab N.A., Amro B. (2020). Parental Smoking Behavior and Childhood Cancer: A Case-control Study. Am. J. Health Behav..

[116] Frederiksen L.E., Erdmann F., Wesseling C., Winther J.F., Mora A.M. (2020). Parental tobacco smoking and risk of childhood leukemia in Costa Rica: A population-based case-control study. Environ. Res..

[117] Doganis D., Katsimpris A., Panagopoulou P., Bouka P., Bouka E., Moschovi M., Polychronopoulou S., Papakonstantinou E., Tragiannidis A., Katzilakis N. (2020). Maternal lifestyle characteristics and Wilms tumor risk in the offspring: A systematic review and meta-analysis. Cancer Epidemiol..

[118] Medina-Sanson A., Núñez-Enríquez J.C., Hurtado-Cordova E., Pérez-Saldivar M.L., Martínez-García A., Jiménez-Hernández E., Fernández-López J.C., Martín-Trejo J.A., Pérez-Lorenzana H., Flores-Lujano J. (2020). Genotype-Environment Interaction Analysis of NQO1, CYP2E1, and NAT2 Polymorphisms and the Risk of Childhood Acute Lymphoblastic Leukemia: A Report From the Mexican Interinstitutional Group for the Identification of the Causes of Childhood Leukemia. Front. Oncol..

[119] Cao Y., Lu J., Lu J. (2020). Paternal Smoking Before Conception and During Pregnancy Is Associated With an Increased Risk of Childhood Acute Lymphoblastic Leukemia: A Systematic Review and Meta-Analysis of 17 Case-Control Studies. J. Pediatr. Hematol. Oncol..

[120] Dong C., Wang M., Zhang J., Zhang R., Liu X., Zheng Z., Yang L. (2019). Tobacco smoke exposure and the risk of childhood acute lymphoblastic leukemia and acute myeloid leukemia: A meta-analysis. Medicine.

[121] Rios P., Bailey H.D., Poulalhon C., Valteau-Couanet D., Schleiermacher G., Bergeron C., Petit A., Defachelles A.-S., Marion G., Sirvent N. (2019). Parental smoking, maternal alcohol consumption during pregnancy and the risk of neuroblastoma in children. A pooled analysis of the ESCALE and ESTELLE French studies. Int. J. Cancer.

[122] Kessous R., Wainstock T., Sheiner E. (2019). Smoking during pregnancy as a possible risk factor for pediatric neoplasms in the offspring: A population-based cohort study. Addict. Behav..

[123] Milne E., Greenop K.R., Petridou E., Bailey H.D., Orsi L., Kang A.Y., Baka M., Bonaventure A., Kourti M., Metayer C. (2018). Maternal consumption of coffee and tea during pregnancy and risk of childhood ALL: A pooled analysis from the childhood Leukemia International Consortium. Cancer Causes Control CCC.

[124] de Smith A.J., Kaur M., Gonseth S., Endicott A., Selvin S., Zhang L., Roy R., Shao X., Hansen H.M., Kang A.Y. (2017). Correlates of Prenatal and Early-Life Tobacco Smoke Exposure and Frequency of Common Gene Deletions in Childhood Acute Lymphoblastic Leukemia. Cancer Res..

[125] Tettamanti G., Ljung R., Mathiesen T., Schwartzbaum J., Feychting M. (2016). Maternal smoking during pregnancy and the risk of childhood brain tumors: Results from a Swedish cohort study. Cancer Epidemiol..

[126] Vienneau D., Infanger D., Feychting M., Schüz J., Schmidt L.S., Poulsen A.H., Tettamanti G., Klæboe L., Kuehni C.E., Tynes T. (2016). A multinational case-control study on childhood brain tumours, anthropogenic factors, birth characteristics and prenatal exposures: A validation of interview data. Cancer Epidemiol..

[127] Azary S., Ganguly A., Bunin G.R., Lombardi C., Park A.S., Ritz B., Heck J.E. (2016). Sporadic Retinoblastoma and Parental Smoking and Alcohol Consumption before and after Conception: A Report from the Children’s Oncology Group. PLoS ONE.

[128] Metayer C., Petridou E., Aranguré J.M.M., Roman E., Schüz J., Magnani C., Mora A.M., Mueller B.A., de Oliveira M.S.P., Dockerty J.D. (2016). Parental Tobacco Smoking and Acute Myeloid Leukemia: The Childhood Leukemia International Consortium. Am. J. Epidemiol..

[129] Chu P., Wang H., Han S., Jin Y., Lu J., Han W., Shi J., Guo Y., Ni X. (2016). Maternal smoking during pregnancy and risk of childhood neuroblastoma: Systematic review and meta-analysis. J. Cancer Res. Ther..

[130] Orsi L., Rudant J., Ajrouche R., Leverger G., Baruchel A., Nelken B., Pasquet M., Michel G., Bertrand Y., Ducassou S. (2015). Parental smoking, maternal alcohol, coffee and tea consumption during pregnancy, and childhood acute leukemia: The ESTELLE study. Cancer Causes Control CCC.

[131] Momen N.C., Olsen J., Gissler M., Li J. (2016). Exposure to maternal smoking during pregnancy and risk of childhood cancer: A study using the Danish national registers. Cancer Causes Control CCC.

[132] Mattioli S., Farioli A., Legittimo P., Miligi L., Benvenuti A., Ranucci A., Salvan A., Rondelli R., Magnani C., on behalf of the SETIL Study Group (2014). Tobacco smoke and risk of childhood acute non-lymphocytic leukemia: Findings from the SETIL study. PLoS ONE.

[133] Grufferman S., Lupo P.J., Vogel R.I., Danysh H.E., Erhardt E.B., Ognjanovic S. (2014). Parental military service, agent orange exposure, and the risk of rhabdomyosarcoma in offspring. J. Pediatr..

[134] Farioli A., Legittimo P., Mattioli S., Miligi L., Benvenuti A., Ranucci A., Salvan A., Rondelli R., Conter V., Magnani C. (2014). Tobacco smoke and risk of childhood acute lymphoblastic leukemia: Findings from the SETIL case-control study. Cancer Causes Control CCC.

[135] Greenop K.R., Miller M., Attia J., Ashton L.J., Cohn R., Armstrong B.K., Milne E. (2014). Maternal consumption of coffee and tea during pregnancy and risk of childhood brain tumors: Results from an Australian case-control study. Cancer Causes Control CCC.

[136] Huang Y., Huang J., Lan H., Zhao G., Huang C. (2014). A meta-analysis of parental smoking and the risk of childhood brain tumors. PLoS ONE.

[137] Barrington-Trimis J.L., Searles Nielsen S., Preston-Martin S., Gauderman W.J., Holly E.A., Farin F.M., Mueller B.A., McKean-Cowdin R. (2013). Parental smoking and risk of childhood brain tumors by functional polymorphisms in polycyclic aromatic hydrocarbon metabolism genes. PLoS ONE.

[138] Metayer C., Zhang L., Wiemels J.L., Bartley K., Schiffman J., Ma X., Aldrich M.C., Chang J.S., Selvin S., Fu C.H. (2013). Tobacco smoke exposure and the risk of childhood acute lymphoblastic and myeloid leukemias by cytogenetic subtype. Cancer Epidemiol. Biomark. Prev. Publ. Am. Assoc. Cancer Res. Cosponsored Am. Soc. Prev. Oncol..

[139] Milne E., Greenop K.R., Scott R.J., Ashton L.J., Cohn R.J., de Klerk N.H., Lyon J.L., Swanson G.M., Weiss N.S., West D. (2013). Parental smoking and risk of childhood brain tumors. Int. J. Cancer.

[140] Rossides M., Kampitsi C.E., Talbäck M., Mogensen H., Wiebert P., Feychting M., Tettamanti G. (2022). Risk of Cancer in Children of Parents Occupationally Exposed to Hydrocarbon Solvents and Engine Exhaust Fumes: A Register-Based Nested Case-Control Study from Sweden [1960–2015]. Environ. Health Perspect..

[141] Zhang Y., Chen D., Shi R., Kamijima M., Sakai K., Tian Y., Gao Y. (2021). Indoor volatile organic compounds exposures and risk of childhood acute leukemia: A case-control study in shanghai. J. Environ. Sci. Health Part A Tox Hazard. Subst. Environ. Eng..

[142] Stayner L.T., Schullehner J., Semark B.D., Jensen A.S., Trabjerg B.B., Pedersen M., Olsen J., Hansen B., Ward M.H., Jones R.R. (2021). Exposure to nitrate from drinking water and the risk of childhood cancer in Denmark. Environ. Int..

[143] Hvidtfeldt U.A., Erdmann F., Urhøj S.K., Brandt J., Geels C., Ketzel M., Frohn L.M., Christensen J.H., Sørensen M., Raaschou-Nielsen O. (2020). Air pollution exposure at the residence and risk of childhood cancers in Denmark: A nationwide register-based case-control study. eClinicalMedicine.

[144] Wang Y., Gao P., Liang G., Zhang N., Wang C., Wang Y., Nie L., Lv X., Li W., Guo Q. (2019). Maternal prenatal exposure to environmental factors and risk of childhood acute lymphocytic leukemia: A hospital-based case-control study in China. Cancer Epidemiol..

[145] Raaschou-Nielsen O., Hvidtfeldt U.A., Roswall N., Hertel O., Poulsen A.H., Sørensen M. (2018). Ambient benzene at the residence and risk for subtypes of childhood leukemia, lymphoma and CNS tumor. Int. J. Cancer.

[146] Ghosh S., Loffredo C.A., Mitra P.S., Trnovec T., Palkovicova Murinova L., Sovcikova E., Hoffman E.P., Makambi K.H., Dutta S.K. (2018). PCB exposure and potential future cancer incidence in Slovak children: An assessment from molecular finger printing by Ingenuity Pathway Analysis [IPA®] derived from experimental and epidemiological investigations. Environ. Sci. Pollut. Res. Int..

[147] Park A.S., Ritz B., Ling C., Cockburn M., Heck J.E. (2017). Exposure to ambient dichloromethane in pregnancy and infancy from industrial sources and childhood cancers in California. Int. J. Hyg. Environ. Health.

[148] Whitehead T.P., Adhatamsoontra P., Wang Y., Arcolin E., Sender L., Selvin S., Metayer C. (2017). Home remodeling and risk of childhood leukemia. Ann. Epidemiol..

[149] Metayer C., Scelo G., Kang A.Y., Gunier R.B., Reinier K., Lea S., Chang J.S., Selvin S., Kirsch J., Crouse V. (2016). A task-based assessment of parental occupational exposure to organic solvents and other compounds and the risk of childhood leukemia in California. Environ. Res..

[150] Jiang W.C., Wu S.Y., Ke Y.B. (2016). Association of exposure to environmental chemicals with risk of childhood acute lymphocytic leukemia. Chin. J. Prev. Med..

[151] Bailey H.D., Metayer C., Milne E., Petridou E.T., Infante-Rivard C., Spector L.G., Clavel J., Dockerty J.D., Zhang L., Armstrong B.K. (2015). Home paint exposures and risk of childhood acute lymphoblastic leukemia: Findings from the Childhood Leukemia International Consortium. Cancer Causes Control CCC.

[152] Ward M.H., Colt J.S., Deziel N.C., Whitehead T.P., Reynolds P., Gunier R.B., Nishioka M., Dahl G.V., Rappaport S.M., Buffler P.A. (2014). Residential levels of polybrominated diphenyl ethers and risk of childhood acute lymphoblastic leukemia in California. Environ. Health Perspect..

[153] Gao Y., Zhang Y., Kamijima M., Sakai K., Khalequzzaman M., Nakajima T., Shi R., Wang X., Chen D., Ji X. (2014). Quantitative assessments of indoor air pollution and the risk of childhood acute leukemia in Shanghai. Environ. Pollut. Barking Essex.

[154] Parodi S., Merlo D.F., Ranucci A., Miligi L., Benvenuti A., Rondelli R., Magnani C., Haupt R., SETIL Working Group (2014). Risk of neuroblastoma, maternal characteristics and perinatal exposures: The SETIL study. Cancer Epidemiol..

[155] Peters S., Glass D.C., Greenop K.R., Armstrong B.K., Kirby M., Milne E., Fritschi L. (2014). Childhood brain tumours: Associations with parental occupational exposure to solvents. Br. J. Cancer.

[156] Ruckart P.Z., Bove F.J., Maslia M. (2013). Evaluation of exposure to contaminated drinking water and specific birth defects and childhood cancers at Marine Corps Base Camp Lejeune, North Carolina: A case-control study. Environ. Health Glob. Access Sci. Source.

[157] Shi R., Gao Y., Zhang Y., Gao Y.J., Zhu S., Wang X.J., Jin P., Tian Y. (2013). Relationship between parental exposure to chemicals and risk of childhood acute leukemia. Chin. J. Ind. Hyg. Occup. Dis..

[158] Brabant C., Geerinck A., Beaudart C., Tirelli E., Geuzaine C., Bruyère O. (2022). Exposure to magnetic fields and childhood leukemia: A systematic review and meta-analysis of case-control and cohort studies. Rev. Environ. Health.

[159] Amoon A.T., Swanson J., Magnani C., Johansen C., Kheifets L. (2022). Pooled analysis of recent studies of magnetic fields and childhood leukemia. Environ. Res..

[160] Seomun G., Lee J., Park J. (2021). Exposure to extremely low-frequency magnetic fields and childhood cancer: A systematic review and meta-analysis. PLoS ONE.

[161] Núñez-Enríquez J.C., Correa-Correa V., Flores-Lujano J., Pérez-Saldivar M.L., Jiménez-Hernández E., Martín-Trejo J.A., Espinoza-Hernández L.E., Medina-Sanson A., Cárdenas-Cardos R., Flores-Villegas L.V. (2020). Extremely Low-Frequency Magnetic Fields and the Risk of Childhood B-Lineage Acute Lymphoblastic Leukemia in a City With High Incidence of Leukemia and Elevated Exposure to ELF Magnetic Fields. Bioelectromagnetics.

[162] Crespi C.M., Swanson J., Vergara X.P., Kheifets L. (2019). Childhood leukemia risk in the California Power Line Study: Magnetic fields versus distance from power lines. Environ. Res..

[163] Auger N., Bilodeau-Bertrand M., Marcoux S., Kosatsky T. (2019). Residential exposure to electromagnetic fields during pregnancy and risk of child cancer: A longitudinal cohort study. Environ. Res..

[164] Talibov M., Olsson A., Bailey H., Erdmann F., Metayer C., Magnani C., Petridou E., Auvinen A., Spector L., Clavel J. (2019). Parental occupational exposure to low-frequency magnetic fields and risk of leukaemia in the offspring: Findings from the Childhood Leukaemia International Consortium [CLIC]. Occup. Environ. Med..

[165] Amoon A.T., Crespi C.M., Ahlbom A., Bhatnagar M., Bray I., Bunch K.J., Clavel J., Feychting M., Hémon D., Johansen C. (2018). Proximity to overhead power lines and childhood leukaemia: An international pooled analysis. Br. J. Cancer.

[166] Su L., Zhao C., Jin Y., Lei Y., Lu L., Chen G. (2018). Association between parental occupational exposure to extremely low frequency magnetic fields and childhood nervous system tumors risk: A meta-analysis. Sci. Total Environ..

[167] Kheifets L., Crespi C.M., Hooper C., Cockburn M., Amoon A.T., Vergara X.P. (2017). Residential magnetic fields exposure and childhood leukemia: A population-based case-control study in California. Cancer Causes Control CCC.

[168] Su L., Fei Y., Wei X., Guo J., Jiang X., Lu L., Chen G. (2016). Associations of parental occupational exposure to extremely low-frequency magnetic fields with childhood leukemia risk. Leuk. Lymphoma.

[169] Crespi C.M., Vergara X.P., Hooper C., Oksuzyan S., Wu S., Cockburn M., Kheifets L. (2016). Childhood leukaemia and distance from power lines in California: A population-based case-control study. Br. J. Cancer.

[170] Bunch K.J., Swanson J., Vincent T.J., Murphy M.F.G. (2016). Epidemiological study of power lines and childhood cancer in the UK: Further analyses. J. Radiol. Prot. Off. J. Soc. Radiol. Prot..

[171] Dechent D., Driessen S. (2016). Re: Role of Electromagnetic Field Exposure in Childhood Acute Lymphoblastic Leukemia and No Impact of Urinary Alpha- Amylase—A Case Control Study in Tehran, Iran. Asian Pac. J. Cancer Prev. APJCP.

[172] Tabrizi M.M., Bidgoli S.A. (2015). Increased risk of childhood acute lymphoblastic leukemia [ALL] by prenatal and postnatal exposure to high voltage power lines: A case control study in Isfahan, Iran. Asian Pac. J. Cancer Prev. APJCP.

[173] Bunch K.J., Swanson J., Vincent T.J., Murphy M.F.G. (2015). Magnetic fields and childhood cancer: An epidemiological investigation of the effects of high-voltage underground cables. J. Radiol. Prot. Off. J. Soc. Radiol. Prot..

[174] Pedersen C., Johansen C., Schüz J., Olsen J.H., Raaschou-Nielsen O. (2015). Residential exposure to extremely low-frequency magnetic fields and risk of childhood leukaemia, CNS tumour and lymphoma in Denmark. Br. J. Cancer.

[175] Salvan A., Ranucci A., Lagorio S., Magnani C., SETIL Research Group (2015). Childhood leukemia and 50 Hz magnetic fields: Findings from the Italian SETIL case-control study. Int. J. Environ. Res. Public Health.

[176] Bunch K.J., Keegan T.J., Swanson J., Vincent T.J., Murphy M.F.G. (2014). Residential distance at birth from overhead high-voltage powerlines: Childhood cancer risk in Britain 1962–2008. Br. J. Cancer.

[177] Zhao L., Liu X., Wang C., Yan K., Lin X., Li S., Bao H., Liu X. (2014). Magnetic fields exposure and childhood leukemia risk: A meta-analysis based on 11,699 cases and 13,194 controls. Leuk. Res..

[178] Pedersen C., Bräuner E.V., Rod N.H., Albieri V., Andersen C.E., Ulbak K., Hertel O., Johansen C., Schüz J., Raaschou-Nielsen O. (2014). Distance to high-voltage power lines and risk of childhood leukemia—An analysis of confounding by and interaction with other potential risk factors. PLoS ONE.

[179] Sermage-Faure C., Demoury C., Rudant J., Goujon-Bellec S., Guyot-Goubin A., Deschamps F., Hemon D., Clavel J. (2013). Childhood leukaemia close to high-voltage power lines—The Geocap study, 2002–2007. Br. J. Cancer.

[180] Ngoc L.T.N., Park D., Lee Y.C. (2022). Human Health Impacts of Residential Radon Exposure: Updated Systematic Review and Meta-Analysis of Case-Control Studies. Int. J. Environ. Res. Public Health.

[181] Moon J., Yoo H. (2021). Residential radon exposure and leukemia: A meta-analysis and dose-response meta-analyses for ecological, case-control, and cohort studies. Environ. Res..

[182] Lu Y., Liu L., Chen Q., Wei J., Cao G., Zhang J. (2020). Domestic radon exposure and risk of childhood leukemia: A meta-analysis. J. BUON Off. J. Balk. Union. Oncol..

[183] Nikkilä A., Arvela H., Mehtonen J., Raitanen J., Heinäniemi M., Lohi O., Auvinen A. (2020). Predicting residential radon concentrations in Finland: Model development, validation, and application to childhood leukemia. Scand. J. Work Environ. Health.

[184] Chen J., Xie L. (2019). Domestic radon exposure and childhood leukaemia and lymphoma: A population-based study in canada. Radiat. Prot. Dosimetry.

[185] Peckham E.C., Scheurer M.E., Danysh H.E., Lubega J., Langlois P.H., Lupo P.J. (2015). Residential Radon Exposure and Incidence of Childhood Lymphoma in Texas, 1995–2011. Int. J. Environ. Res. Public Health.

[186] Del Risco Kollerud R., Blaasaas K.G., Claussen B. (2014). Risk of leukaemia or cancer in the central nervous system among children living in an area with high indoor radon concentrations: Results from a cohort study in Norway. Br. J. Cancer.

[187] Hauri D., Spycher B., Huss A., Zimmermann F., Grotzer M., von der Weid N., Weber D., Spoerri A., Kuehni C.E., Röösli M. (2013). Domestic radon exposure and risk of childhood cancer: A prospective census-based cohort study. Environ. Health Perspect..

[188] Onyije F.M., Olsson A., Baaken D., Erdmann F., Stanulla M., Wollschläger D., Schüz J. (2022). Environmental Risk Factors for Childhood Acute Lymphoblastic Leukemia: An Umbrella Review. Cancers.

[189] https://www.iarc.who.int/wp-content/uploads/2018/07/pr221_E.pdf.

[190] https://monographs.iarc.who.int/wp-content/uploads/2018/06/mono109-F07.pdf.

[191] https://monographs.iarc.who.int/wp-content/uploads/2018/06/mono100F-24.pdf.

[192] McHale C.M., Zhang L., Smith M.T. (2012). Current understanding of the mechanism of benzene-induced leukemia in humans: Implications for risk assessment. Carcinogenesis.

[193] Pech K., Pérez-Herrera N., Vértiz-Hernández Á.A., Lajous M., Farías P. (2023). Health Risk Assessment in Children Occupationally and Para-Occupationally Exposed to Benzene Using a Reverse-Translation PBPK Model. Int. J. Environ. Res. Public Health.

[194] Gruľová D., Caputo L., Elshafie H.S., Baranová B., De Martino L., Sedlák V., Gogaľová Z., Poráčová J., Camele I., De Feo V. (2020). Thymol Chemotype Origanum vulgare L. Essential Oil as a Potential Selective Bio-Based Herbicide on Monocot Plant Species. Molecules.

[195] Ottenbros I., Lebret E., Huber C., Lommen A., Antignac J.P., Čupr P., Šulc L., Mikeš O., Szigeti T., Középesy S. (2023). Assessment of exposure to pesticide mixtures in five European countries by a harmonized urinary suspect screening approach. Int. J. Hyg. Environ. Health.

[196] https://www.iarc.who.int/wp-content/uploads/2018/07/pr236_E.pdf.

[197] Navarrete-Meneses M.d.P., Pérez-Vera P. (2019). Pyrethroid pesticide exposure and hematological cancer: Epidemiological, biological and molecular evidence. Rev. Environ. Health.

[198] Navarrete-Meneses M.d.P., Salas-Labadía C., Juárez-Velázquez M.d.R., Moreno-Lorenzana D., Gómez-Chávez F., Olaya-Vargas A., Pérez-Vera P. (2023). Exposure to Insecticides Modifies Gene Expression and DNA Methylation in Hematopoietic Tissues In Vitro. Int. J. Mol. Sci..

[199] Nicolella H.D., de Assis S. (2022). Epigenetic Inheritance: Intergenerational Effects of Pesticides and Other Endocrine Disruptors on Cancer Development. Int. J. Mol. Sci..

[200] https://www.who.int/europe/news/item/03-02-2021-world-cancer-day-know-the-facts-tobacco-and-alcohol-both-cause-cancer#:~:text=People%20who%20use%20both%20alcohol,up%20to%2030%20times%20higher.

[201] https://monographs.iarc.who.int/wp-content/uploads/2018/06/mono100E-6.pdf.

[202] NCI (2022). Electromagnetic Fields and Cancer. https://www.cancer.gov/about-cancer/causes-prevention/risk/radiation/electromagnetic-fields-fact-sheet.

[203] Schüz J., Erdmann F. (2016). Environmental Exposure and Risk of Childhood Leukemia: An Overview. Arch. Med. Res..

[204] Onyije F.M., Olsson A., Bouaoun L., Schüz J. (2023). Synthesized evidence for childhood acute lymphoblastic leukemia. Front. Pediatr..

